# A case study of physical and social barriers to hygiene and child growth in remote Australian Aboriginal communities

**DOI:** 10.1186/1471-2458-9-346

**Published:** 2009-09-18

**Authors:** Elizabeth McDonald, Ross Bailie, Jocelyn Grace, David Brewster

**Affiliations:** 1 Menzies School of Health Research, Institute of Advanced Studies, Charles Darwin University, Darwin, Australia; 2 National Drug Research Institute, Curtin University of Technology, Perth, Western Australia, Australia; 3 School of Medicine, James Cook University, Cairns, Australia

## Abstract

**Background:**

Despite Australia's wealth, poor growth is common among Aboriginal children living in remote communities. An important underlying factor for poor growth is the unhygienic state of the living environment in these communities. This study explores the physical and social barriers to achieving safe levels of hygiene for these children.

**Methods:**

A mixed qualitative and quantitative approach included a community level cross-sectional housing infrastructure survey, focus groups, case studies and key informant interviews in one community.

**Results:**

We found that a combination of crowding, non-functioning essential housing infrastructure and poor standards of personal and domestic hygiene underlie the high burden of infection experienced by children in this remote community.

**Conclusion:**

There is a need to address policy and the management of infrastructure, as well as key parenting and childcare practices that allow the high burden of infection among children to persist. The common characteristics of many remote Aboriginal communities in Australia suggest that these findings may be more widely applicable.

## Background

Internationally, Indigenous people tend to have relatively poor living conditions and health status compared to the general population of the countries in which they live. This is associated with experiencing disadvantage across the generally recognised social determinants of health, as well as the consequences of acculturation and the loss of cultural cohesion [1]. Indigenous children are particularly vulnerable to experiencing malnutrition and infection that not only affects their growth and development, but also their cognitive development, educational outcomes and health and wellbeing throughout life [2,3]. For Australian Aboriginal children living in remote communities the situation is particularly acute. These children experience relatively high rates of poor growth, common childhood infections [4] and serious diseases such as acute rheumatic fever, rheumatic heart disease, and trachoma [5-9] compared to their non-Aboriginal peers, to Indigenous children living in North America and New Zealand, and to children from some developing countries [6,10,11]. In 2006, Indigenous (Aboriginal and Torres Strait Islander) post-neonate infants (infants aged between 28 days and 12 months of age) in the Northern Territory (NT) were five times more likely to be admitted to hospital than NT non-Indigenous post-neonates[4]. Co-morbidity was common with the average number of conditions associated with each admission being 3.2 [4]. Diseases of the respiratory system (especially acute bronchitis and bronchiolitis), infectious and parasitic diseases (especially intestinal infections), diseases of the skin (especially scabies) and nutritional diseases (especially anaemia and malnutrition) carried the highest relative risk for admission for Indigenous children compared with non-Indigenous children.

High levels of social, economic and environmental disadvantage underlie the health problems in remote Indigenous communities [7,12]. Despite Australia's wealth, living conditions in these communities are poor. This is highlighted by survey data on Aboriginal housing in the NT which showed that only between 38% and 69% of houses had all the functioning components required to effectively conduct each of six key 'healthy living practices'. Healthy living practices include: wash people (54%), wash clothes (68%), functioning toilet (55%), remove waste water (61%), remove waste rubbish (69%) and prepare and store food (38%) [13]. Poor housing conditions lead to unsanitary environments and an increase in infections, especially enteric conditions. Household crowding is common and Aboriginal community councils struggle to maintain their existing housing stock in a satisfactory condition.

The barriers to achieving safe levels of hygiene in this context are many and varied. However, there has been little research into these barriers. This study explored the physical and social barriers to achieving safe levels of hygiene for Australian Aboriginal people living in remote communities in the NT. In particular, we investigated the barriers to meeting the hygiene needs of young children.

## Methods

This research took place in a remote Aboriginal community located in north-western Arnhem Land between May 2002 and May 2005. It was a descriptive study using mixed methods. The study consists of two discrete but linked components (Components A and B). Data collection was by either tape-recording or taking handwritten notes of interviews and discussions, as dictated by the preference of participants. The NT Department of Health and Community Services and Menzies School of Health Research (MSHR) Human Research Ethics Committee, and the local Aboriginal governing body of the community concerned provided approval for this research. Everyone who participated in the study provided written consent.

### The Setting

The study community has evolved from a base used by a group of non-Indigenous buffalo hunters in the late 19^th ^Century. Early in the 20^th ^Century, the government took over the site to use as an experimental dairy farm. From 1925 to the mid 1970s, it was a mission station. The community became self-governing in the 1970s and this was the case at the time of the study.

From a population of less than 20 people in 1925 (when the mission station was first established), the population is now estimated to be between 854 [14] and 1100 [15] persons. In 2001, 37.5% of the total Indigenous population in this community were aged less than 15 years, and of this number 49% were less than four years of age [14]. The community operates administratively in English, and the vast majority of people can speak and understand basic English language [16].

The services available in the study community mirror those available in most remote Australian Aboriginal communities. They include a store, health centre, police station, primary school, art centre, garage, social club, bank, and social welfare and post office agencies. At the time of the study, the community council, through grant-in-aid funding, provided some additional community services, for example, in the area of aged care and a women's resource centre. The community council administered the construction, repairs, and maintenance of community-housing, also environmental health programs including garbage collection and disposal, animal control and the maintenance of public places. Essential services available to the community include electricity (the community has its own generator), piped bore water, sewerage (pond) system, airstrip and telephone communications. These services remain the responsibility of the relevant government and private agencies, while the community council is responsible for ensuring day-to-day service provision.

The pattern of poor health experienced by people who live in the study community was, and remains, consistent with most remote Indigenous communities in Northern Australia. Community members experience high rates of diabetes, renal disease and respiratory disease [17]. This general poor health state results in a reduced life expectancy [18]. Children's health is also poor [4,19,20]. In 2003, in the study community, approximately 30% of the children aged <5 years were categorised as underweight [21], that is their growth rates were <-2 Standard Deviations Weight for Age [22].

The health centre offered a comprehensive range of primary health care services. At the time of the study, four registered nurses, three Aboriginal health workers and one medical officer provided these services. However, due to recruitment difficulties, the centre was often understaffed and can be without a resident medical officer for extended periods. High staff turnover impeded the development of trusting relationships between community members and staff. Achieving continuity of care and program effectiveness under these circumstances was challenging.

Most community members have only a low level of numeracy and literacy, with most people only having attended the community school to eighth or ninth grade. In general, school attendance by primary school aged children in the community was poor [18]. Very few adolescents left the community to attend high school in Darwin.

All housing in the community was public housing and not dissimilar in design to public housing available in urban centres in the NT. Houses largely consisted of a kitchen, living room, laundry, toilet, bathroom and three bedrooms and have all essential utilities (water, power, sewerage or septic) available. The standard of houses in the study community was variable. Some houses - built over 40 years ago, have been the subject of many renovations. Generally, the design and standard of these older houses is poor. Other more recently built houses are of an improved design and provide better facilities. The outward appearance of most houses was generally poor. Many houses had missing or broken insect screens and windows, and with litter present in the yard. The community council housing repairs and maintenance program functioned at a low level over the time of the study, providing only a limited service. The construction of houses has not kept pace with population growth and crowding was an ongoing problem.

The level of disadvantage experienced by people living in the study community remained high. The economy of the community was dependent on tied government grant funding and social welfare payments. Most community members were 'employed' on a part-time basis by the community council under the Community Development Employment Program (CDEP), a work-for-the-dole scheme. The total income most families received was the same amount as paid to recipients of unemployment benefits.

The study community was a suitable site for this research because its location, climate, population size, and health profile reflect characteristics of the sort of communities inhabited by many Aboriginal people in northern Australia [17,23-25].

### Component A - Housing, Crowding and Hygiene

This component investigated the factors of housing, crowding and hygiene as these might contribute to poor child health outcomes in the community. The aim of Component A was to provide a better understanding of the extent to which the risk factors of poor housing, crowding and a low standard of hygiene exist in the study community, as well as to identify the likely reasons for non-functional infrastructure and high levels of environmental contamination in houses. Data are from two sources, a community level cross-sectional housing infrastructure survey and focus group discussions.

#### Cross Sectional Survey

The 2002 Indigenous Housing Association of the Northern Territory (IHANT) and MSHR Housing Infrastructure and Child Health (HICH) Pilot Study [26] used three data collection methods including a housing infrastructure survey, interviewer administered questionnaires for householders and carers of children under seven years, and an audit of health records of children under seven years. Data on the houses where children under seven years lived (n = 47) were extracted from the 2002 Pilot Study cross-sectional survey, then descriptive analysis completed.

Bi-annual housing infrastructure surveys were part of the existing government Indigenous housing program. The inspection checklist provided for surveyors to mark the condition of each item of infrastructure according to five defined coding categories [see Additional file 1]. This study reports on the three categories that are likely to impede members of households from easily practising good hygiene and are likely to contribute to poor environmental hygiene. These categories include, 'major problem' - the item requires repair, otherwise, it will affect the health or safety of the tenants; 'urgent problem' - a health and safety issue is present; 'missing' - an item is not present but is urgently needed.

The indicators of environmental contamination used were the presence or absence of contaminants such as faeces, including soiled nappies, or other decaying organic matter such as meat or other food remnants easily visible on surfaces inside, or on any concrete surfaces surrounding the house. The potential level of risk posed to children's health by poor domestic and environmental hygiene was then assessed with reference to the common childhood infections endemic in the community, and according to the factors described by the International Scientific Forum on Home Hygiene [27].

#### Focus Group

With the assistance of environmental health and technical officers who work in the remote Aboriginal housing sector, a list of nine reasons for why items of housing infrastructure are likely to need repairs or be missing were identified. The reasons included 'normal wear and tear', 'inappropriate use', 'neglect', 'inappropriate technology', 'poor construction/incorrect installation', 'general damage', 'malicious damage', 'more than one cause' and 'indeterminate'. Definitions for each of these categories were developed [see Additional file 1]. The three government officers who completed the 2002 Pilot Study housing survey participated in the focus group. The focus group process included the moderator giving the completed housing survey form to the officer who had conducted the inspection, as well as some additional information taken from the householder questionnaire about the house. The officer was first asked to choose from the list of categories what he/she considered was the primary reason that an item needed repair or was missing, and then to nominate an alternative (next most likely) reason and to discuss the rationale for their decisions. Themes that reoccurred and the implications of these were taken into account when determining findings.

### Component B - Health and Hygiene Knowledge

**Figure 1:**
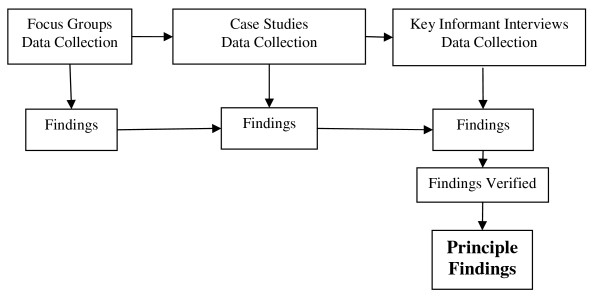
**Overview of study process and process followed to identify study findings**.

This component identified community members' level of knowledge about hygiene and hygiene behaviour. The aim of the second component was to assess community members' level of knowledge about the transmission mechanisms of common childhood infections, as well as their attitudes towards the hygiene behaviours needed to reduce the transmission of these infections. Study participants were recruited from three sections of the community and three different data collection methodologies were used. The methodologies included focus groups, case studies and key informant interviews. Correspondingly, the three categories of participants recruited were men and women likely to have lower levels of education, carers whose children experienced satisfactory levels of growth in the first 12 months of life, and men and women considered to have higher levels of education or who held responsible positions in the community. In addition, among the key-informants were two non-Aboriginal persons in key positions who had lived in the community for extended periods. Only after taking into consideration the data collected from all the study participants were findings identified (Figure 1). For the purpose of this study, the definition of 'attitudes' was the views formed based on the beliefs or feelings held about the consequences of performing behaviours, that is whether those consequences are valued or not [28,29]. The themes underpinning data collection were those that emerged from an examination of the local epidemiological data [19] and literature on environmental and behavioural factors which increase transmission of infection among children [30].

#### Focus Groups

The focus groups conducted utilised the principles of the '3 pile sorting cards' participatory research methodology [31,32]. An artist created drawings that represented common community scenes [see Additional file 1] that included good and poor hygiene practices, for example, women hanging washed clothes out to dry and young children defaecating in the open.

Those invited to participate in focus groups were adults in the community most likely to have the lowest level of formal education, such as manual workers and traditional artists. Twenty-eight (28) people (19 women and 9 men) participated in nine focus group sessions, their ages ranged from 14 years to approximately 50 years. Group sizes ranged from two to five persons and were sex specific, with three male and six female groups. Traditionally, Aboriginal societies in the NT observed strict sexual differentiation and rules of avoidance between those of the opposite sex in particular kin relations, and these persist to date. Asking men and women to sit together and discuss the subjects of interest in this study would be inappropriate and would inhibit open discussion.

For all focus group sessions the lead researcher, a person who had previously lived and worked in this and other remote Aboriginal communities as a remote area nurse acted as note taker and observer. A second researcher, also experienced in working in remote Aboriginal acted as moderator. A protocol was prepared to introduce and explain the process to participants [see Additional file 1]. To maintain a non-threatening environment, the atmosphere was kept light and the process made as enjoyable as possible. Extra care was taken to depersonalise issues under discussion, including when asking questions to clarify points. Questions were put to the group as a whole, avoiding directly questioning individuals. The focus group process involved participants looking at a picture card and deciding, as a group, if the scenario depicted on the card was 'good', 'not good', or indeterminate (in this study the commonly used local term of 'not sure' was used). Once all the cards were seen, and placed on respective piles, the moderator commenced the second stage of the process. Taking one card at a time she asked the group for the reason why they had placed the card on a specific pile. This process being repeated for each card. Participants were encouraged to respond but the moderator did not subject participants to repeated questioning. If participants were particularly hesitant in making a decision, the moderator offered them the option of 'not sure'. To promote the validity of the data, the moderator and the author reviewed each session's notes and discussed these at the end of each day. The accuracy of the notes was checked, as were aspects of the process for each group, each day.

The focus group data were analysed in a stepped manner [see Additional file 1] as follows:

• collating responses according to each picture card

• comparing levels of knowledge about infection transmission mechanisms and attitudes to hygiene behaviours between those focus groups who viewed the same or similar cards

• grouping the responses according to the three disease themes - skin, diarrhoeal and respiratory disease

• identifying and collating inconsistent and consistent responses

• interpreting what attitudes or level of knowledge about infectious disease transmission mechanisms and some personal hygiene behaviours might be held.

#### Case Studies

Case studies were carried out to determine why some children grow satisfactorily in their first year of life compared to the majority of their peers who experience poor growth. For the purposes of this study, satisfactory growth was said to have occurred if an infant had incremental weight gains to maintain an upward trajectory of their growth curve [see Additional 1]. Attention paid to carers' level of knowledge about, and attitudes to hygiene, their childcare practices and their living environments. Case study participants were selected using a positive deviant approach, having identified primary carers, who, while typically poor, appear to be caring for their children more effectively than others in the community [33].

In November 2002, the growth records for children aged under three years (n = 33) in the study community at the time of the 2002 Pilot Study were reviewed to identify those children whose growth was satisfactory. Children diagnosed with medical conditions likely to impact on their growth, as well as children who had lived away from the community for the greater proportion of their life, were not eligible to be included in the study. Only nine children (27%) had satisfactory growth patterns after six months of age. Four of these children were ineligible to be included in the study; three children had lived in Darwin for extended periods, while one child had received food supplements from birth for medical reasons.

The methods used to collect data included in-depth interviews with the mothers, discussions with the maternal and child health nurse and a review of health centre records and data collected as part of the 2002 Pilot Study on housing, carer and child health. To assess whether the 'deviant' nature of their growth was maintained, the children's weights were reviewed 12 months after the initial audit. The growth patterns of older siblings were examined to see if patterns of satisfactory growth applied to other children having the same carer.

Carer interview data were analysed and grouped according to the themes of knowledge of, and attitudes towards, hygiene behaviors and childcare practices. The analysis was completed in four parts. Initially, a personal profile for each carer was developed. These data were at first explored at individual carer level and then comparisons made between carers. The second level analysis involved determining the continued growth patterns for case study children. Carer interview records were analysed and responses grouped according to the themes of level of knowledge about hygiene, the role of hygiene behaviors, and childcare activities. Factors identified as unusual to a particular carer, for example a higher level of education, were investigated in order to see if this might explain why the case study children had a satisfactory growth rate compared to other children. Key demographic, housing and environmental health factors for each case study child were examined to gauge their overall living circumstances and compare these to the details of the living environments of other young children in the study community.

#### Key informant interviews

Persons employed in positions in the community who indicated they had a higher level of education and/or formal qualifications were invited to participate in the key informant interviews. These were conducted to gain a better understanding of common hygiene related childcare practices in the study community, as well as to clarify and verify information obtained from other sources and the researcher's personal observations. They were also used to identify a broad range of 'attitudes' in the community, and to draw on their professional and/or cultural knowledge and experience for advice in the analysis of the preliminary findings from the focus groups and case studies. Ten key informants (seven Aboriginal and three non-Aboriginals persons) participated.

The staged approach to data analysis for this component of the study consisted of: 1) identifying and grouping information according to levels of knowledge about the transmission of infection, attitudes to hygiene behaviours and other child care practices; 2) comparing these findings with focus group and case study findings; and 3) amalgamating key informant, case study and focus group findings so key themes could be identified and interpreted and principle findings identified. Conclusions were discussed and verified with several Aboriginal and non-Aboriginal key informants.

## Results

### Component A - Housing, Crowding and Hygiene

Forty-one of the 47 houses (89.3%) had one or more items described as needing major or urgent repair or an item considered essential was missing. Among these houses, the primary reasons for household items having major problems (n = 49) were the provision of inappropriate technology (28.6%, n = 14), normal wear and tear (26.5%, n = 13) and inappropriate use (20.4%, n = 10). The primary reasons for household items needing urgent repairs (n = 90) were normal wear and tear (27.7%, n = 25), inappropriate technology (20%, n = 18), general damage (18.8%, n = 17), inappropriate use (14.4%, n = 13) and malicious damage (12.2%, n = 11). Combining the results from these two categories (major problems and urgent repairs) found that normal wear and tear (27.3%, n = 38) was the most common cause of items needing major or urgent repairs. The provision of inappropriate technology ranked second (23%, n = 32). General damage, considered to be caused by residents trying to do repairs themselves, accounted for 13.7% (n = 19) of the urgent or major repairs required. The three government officers who completed the 2002 Pilot Study housing survey believed there were several likely reasons to explain these findings.

Firstly, they considered that at the time of the survey the community did not have the resources available to undertake the timely repair and maintenance of essential items. This meant that the service was unable to be proactive in its activities. After speaking to housing administrators and households, it was clear that there was a vicious cycle. Administrators blamed the households for the large amount of repairs required, and households blamed the administrators for allowing infrastructure to deteriorate. Many households had stopped reporting their repair needs because the waiting time for repair services was so long, or believing that action would not be taken. It appeared that some householders failed to recognise that items of infrastructure required ongoing maintenance to stay in good order, while not reporting problems early (when minor repairs would suffice) was also an issue.

Wear and tear, thought by the focus group participants to be the result of increased usage due to crowding, was found to be responsible for 27.3% (n = 38) of items of infrastructure that needed repairs. The reasons for three toilet cisterns needing repairs included normal wear and tear (2) and general damage (1). The reason for 30 of the 45 taps needing repair was normal wear and tear. Participants considered that a timely repairs and maintenance service would have prevented the need for some repairs and the extent of the problem with others. Faulty or non-functioning infrastructure items considered inappropriate because of design issues or the materials used included hot-water systems (17), kitchen bench tops (10), stove tops (1), ovens (4).

The two categories, 1) the provision of inappropriate essential housing infrastructure items and 2) inappropriate use by householders, somewhat overlap. The first category focuses on the quality of items (workmanship, materials used and design), while the second takes account of householder behaviour. Some might see the former as the primary cause of the problems, while others see householders as primarily responsible.

Inappropriate use of infrastructure items accounted for 16.5% (n = 23) of the repairs needed. The items affected by inappropriate use included stovetops (9), ovens (9), bathroom basins (2), shower drains (2) and toilet pan (1). Examples of the inappropriate use of stovetops by householders included placing heavy cooking pots on the coil elements causing them to break and using elements in place of matches to light cigarettes.

The focus group participants believed that the non-functioning infrastructure items categorised as general damage (13.7%, n = 19) also reflected the absence of a timely and effective repairs and maintenance program in the community. The category 'general damage' was included in response to the concern that some items of infrastructure might be incorrectly identified, based on appearance only, as intentional damage. Participants considered that this type of damage occurred when items became faulty or non-functional and householders' failed attempts to undertake repairs made the problems worse.

Thirteen items were considered to need repair, or were missing, due to malicious or other intentional damage. Householders frequently reported that individuals under the influence of drugs or alcohol, or 'petrol sniffers' caused this type of damage.

Of the items missing from houses (n = 41) but urgently needed, seven houses (17%) did not have a functioning refrigerator, and 17 (41%) did not have a functioning washing machine.

In only two cases (1.4%) was the primary reason for an item needing to be repaired coded as 'indeterminate'. This highlights the confidence the participants had in stating what the primary reason was for items needing repair, as well as the degree of consensus as to the reasons why infrastructure items progressed to have serious problems. The alternative reasons provided generally resulted in the reversal of the options normal wear and tear and use of inappropriate technology, and inappropriate use by residents and general damage.

The results of this study indicate that in the study community household residents, and particularly the young children, live constantly with a medium to high-level risk of acquiring infections from environmental contamination. In 19 (42.2%) of the houses, faeces or other decaying matter such as meat and other food remnants was observed in the immediate living environment. In five (11%) of the houses contaminated matter was observed on surfaces both inside and outside the house.

A significant level of crowding was present in many of the houses with young children. The mean number of persons per bedroom in the houses where children under seven years lived was 3.4. In 38 of the 47 houses, the housing occupancy standard [34] of a maximum of two persons per each available bedroom was breached. However, there was no statistically significant association between the level of crowding and the number of items that needed repairs or missing items.

### Component B - Health and Hygiene Knowledge

#### Focus Groups

##### Knowledge

All groups concurred in their general response to 12 of the 27 scenarios (44.4%) presented on the cards. Of the 12 scenarios, six were considered 'good' and six 'not good'. However, the rationale that underpinned decision-making was not the same across the groups. Having provided a biologically plausible response as to whether a behaviour was 'good' or 'not good', when asked to explain their decision many did so incorrectly, indicating that most focus group participants did not have a good level of understanding about hygiene.

##### Attitudes

The clear consensus in responses to 12 picture cards indicates that common attitudes are held concerning these behaviours, and that 'good' behaviours were seen in a very positive light while the 'not good' behaviours were viewed negatively. When participants were unsure how to categorise a picture they were very thoughtful in giving their responses. They could identify behaviours they considered both 'good' and 'not good' in some scenarios and tried to weigh these up. They preferred to say they were 'unsure' rather than state a behaviour they apparently highly valued was 'not good'. Examples of the drawings shown and the way focus groups categorised the drawings are available in Table S1 [see Additional file 2].

While adults and older children highly valued their privacy and did use toilets when available, participants indicated a very tolerant attitude towards infants and young children defaecating in the open and appeared not to recognise the health risks posed by this practice. For example, no action was taken to remove or cover the faeces of young children. Indeed, many considered the pictures of young children defaecating funny.

##### Skin Infection

The positive effects of regular bathing with soap to prevent skin infections are not well recognised in the study community. However, the image of a clean appearance appears to be highly valued. The health risk posed by young children with respiratory or skin infections sleeping closely was not generally recognised. When some participants did recognise this risk, the social and emotional benefits they associated with this behaviour outweighed any risk.

In general, the mechanisms of cross infection, as this relates to skin infections and infestations, are not well understood. Protective behaviours such as using soap to reduce the number of pathogenic organisms carried on the skin were not mentioned. It did not appear to be understood that clean bedding and clothing reduced the risk of some infections. However, all focus group participants positively reviewed all the pictures conveying images of good hygiene practices and healthy people. Participants did not seem to recognise the disparity of what is shown in the drawings and their own behaviours and living conditions. They indicated no embarrassment, nor did they attempt to justify the difference in what was portrayed in the drawings and their own circumstances. It appeared that the participants did not relate to the scenes in the drawings, rather the scenarios represented ideal situations and something outside the realms of their own lives.

##### Diarrhoeal Disease

Focus group participants did not recognise the health risks associated with the faeces of infants and young children. The practice of young children defaecating in the open was apparently disliked mainly due to the unsightly appearance of the faeces, the smell, and the large number of flies and dogs it attracted. Participants had never seen or heard of a potty, and were unaware of the concept of toilet training children from a young age. The role that flies and dogs have in transmitting infections appeared to be exaggerated in the minds of participants compared to the risks posed to children by contact with soil contaminated by human or dog faeces.

Some participants were familiar with the concept of germs and the role these played in causing disease. However, in general the terms germs, dirt and scabies were used interchangeably with the same inferred meaning. It was not clear if participants believed that germs caused infections or if this response was learnt from past health education activities. Once again, the images of persons undertaking recognised good hygiene practices were positively received. Participants appeared to value the images of good health shown in the pictures illustrating good hygiene practices.

##### Respiratory Disease

The concept that infection is transferred by coughing, or direct contact with nasal discharge, appeared to be understood by many focus group participants. However, the information provided is contradictory in many cases. Some fundamental concepts about the mechanisms by which respiratory infections spread were not understood. While there was a stated awareness that nasal discharge should be wiped from children's faces, participants' reactions to cards, listening to them discussing them, and observing carers with their children indicates that there is a high level of tolerance towards seeing nasal discharge on children's faces.

#### Case Studies

##### The Carers

All the case study children (n = 5) were in the care of their biological mothers and came from socio-economically disadvantaged families. All but one carer finished school before Year 8/9, which is generally at age 13 to 14 years. Despite high rates of smoking among adults in the community, none of the carers smoked and only one said she occasionally drank alcohol.

After considering the circumstances of the carers of case study Children 3, 4 and 5, it appears that the level of support they received from extended family members was central to the manner in which they cared for their children. The carer of case study Child 2 stood out from the rest in that she was living independently. Initially from an Aboriginal community in Central Australia, this carer had attended boarding school in Darwin and completed school to Year 12 (generally at 17 to 18 years). She had part-time employment and lived in a 'staff' house. The carer of Child 2 may be considered as a positive deviant carer due to the manner in which she independently supported and cared for her child, but as she was not originally from the study community, and had left the community before the follow up visit, the impact of these factors could not be further investigated.

##### Continuing Growth Patterns

The centile readings for each child taken from the health centre records in November 2002, and then again in November 2003, showed that only one of the case study children's growth rate followed an upward trajectory for a further 12 months (Table 1). In the case of the other three children, the health records showed that a mix of acute and chronic infections precipitated them loosing or failing to gain weight.

**Table 1:** Centile readings for November 2002 and November 2003

**Child Number**	**Date of Birth**	**Centile Reading**^1^ **November 2002**	**Centile Reading**^1^ **November 2003**
Child 1	26/9/01	70	55
Child 2	21/4/01	45	N/A^2^
Child 3	4/3/02	60	45
Child 4	25/11/01	65	45
Child 5	12/2/01	50	50

^1^Approximate centile range as taken from the growth records of each child. ^2^Mother and child had left the study community.

The health centre records revealed that overall, all five children experienced fewer episodes of scabies, skin infections and diarrhoea than their peers. Up until November 2003 only Child 5 had been diagnosed and treated for scabies, a condition that occurs commonly in this and other remote communities. All the carers stated that their children commonly experienced respiratory infections. Respiratory infections caused the carers the most concern. It was not possible to quantify these episodes of infection due to the difference in the carers' health seeking behaviours and insufficient information was available in the child health records.

##### Knowledge, Attitudes and Behaviour

All the carers maintained a good relationship with the maternal and child health nurse, regularly attending the health centre for immunisation and growth monitoring purposes. They indicated an understanding of the need for growth monitoring and a high level of motivation for their children to grow well.

The carers of Children 1, 2, 3 and 4 were proactive and took measures to prevent their children getting infections, indicating they were aware of some common infection transmission mechanisms. All the carers mentioned they regularly bathed their children using soap to keep them clean, while one mother put a waterproof ground sheet on the dusty ground and she placed her infant down to sit on this. However, all said that they sometimes ran out of soap and soap powder because they had no money. Carers and other community members who took part in this study used the terms "to bath" children or "bathing" children to describe a number of different activities. For example, Child 3's carer said that she used to bath her child in the sink or laundry trough when he was small, but now she baths him under the shower or uses the hose. Bathing can also refer to hosing down or standing children under outdoor taps without the use of soap.

All carers reported that they "washed" their child's bottom after defecation. Four mentioned wiping their child's bottom with paper and disposing of the paper in the toilet. They also mentioned they washed their child's bottom in the bath, shower, laundry trough or outside tap or with the hose. They made no mention of washing their own hands after disposing of their child's soiled nappies or after cleaning faeces from the floor. In the study community, young children are frequently seen naked and playing outside their homes. This practice provides for the comfort of children but makes it difficult to control where these young children defaecate and what measures are taken to clean children's skin and faeces from surfaces afterwards.

The carers of Children 1, 2 and 3 recounted with ease what foods they purchased and provided to their children. However, it is unclear if the carers actually routinely provided the food they described to their children. At the same time, they did exhibit a satisfactory level of knowledge about good nutrition.

All the carers took pride in their children. In particular, the carers of Children 1, 2, 3 and 4 saw themselves as different from the other mothers of small children in the study community. They claimed that many other mothers did not look after their children properly. After prompting, Child 2's carer stated:

I don't want to point the finger, or blame, but a lot of mothers are lazy and playing cards. You see the kids shit near the card ring; kids sitting on the ground, kids put their arms around those mangy dogs and eating dirt. They've probably got bad husbands too.

##### The Living Environment

The condition of houses and the overall picture of the living environment in which the five case study children lived compared favourably with that of many of their peers. Environmental contamination was observed in one of the five houses where the case study children lived. Functioning washing machines and refrigerators were present in all the houses where case study children lived. This is in contrast to the housing survey that showed that 17% of the houses where children under seven years lived had no functioning refrigerator and 41% had no functioning washing machine. The housing occupancy standard of two persons per bedroom was exceeded in all the houses where the case study children lived, however, the crowding index does not provide a true picture of the potential health risks in these houses. For example, in the cases of Children 2, 3 and 4, these children and their siblings slept together with both parents. Child 1 slept with his parents and one sibling while the oldest sibling slept with her grandmother. Child 5 preferred to sleep with her aunty and numerous cousins. Two issues arise from this finding. The first concerns the value of the household crowding index being seen as a determinant of health as opposed to actual sleeping arrangements. Secondly, it became apparent that once mobile and able to express their preferences, young children determine with whom and in what house they would sleep.

#### Key Informant Interviewers

##### Knowledge

Of the seven Aboriginal key informants, only the pre-school teacher and one senior health worker appeared to have more than a superficial knowledge about good hygiene and the transmission mechanisms of infections. Some other Aboriginal key informants initially appeared to have a good level of knowledge but further discussion revealed this not to be the case.

##### Attitudes

A non-Aboriginal key informant believed that community members considered the nasal and purulent ear discharge of many children to be normal. Observing focus group participants with their children confirmed this to be the case. The general appearance and clean faces of four of the case study children indicated that their mothers did not consider nasal discharge as acceptable. However, Child 5 came to sit with her mother during discussions and she had a large amount of tenacious yellow discharge draining from her nose. Her mother, on seeing me notice this, quickly wiped the discharge off with her hand and then wiped her hand on her clothing. The child was not encouraged to blow her nose. The motivation for wiping the child's nose in this instance appeared to be the presence of a non-Aboriginal visitor.

Two non-Aboriginal informants (1 male and 1 female), and one Aboriginal key informant, mentioned that women in the study community were often very tired. A non-Aboriginal key informant said that many women described themselves to him as "giving up." An Aboriginal key informant said:

Mothers get tired, as soon as they sweep and mop others make it dirty straight away again. They leave their plate and knife on the ground - you get sick of picking it up after them all the time.

It was observed, and other key informants reported, that in general adults and older children in the community do not wish to cause young children to get upset. They generally avoid disciplining a child by ignoring his/her behaviour except when the child is in real jeopardy of injuring him/herself. Examples of this behaviour include carers and other adults not being able to prevent young children playing in large muddy puddles made by pigs kept as household pets. Not infrequently, parents report that their sick child did not receive prescribed medicine because "he did not want it".

All the key informants considered the overall standard of hygiene in the study community to be low. However, several pointed out that some individuals were going to great efforts to try to keep their home, themselves and their children clean and healthy.

##### Behaviour

Female Aboriginal key informants were asked which members of the family were responsible for ensuring young children are bathed and taught how to use the toilet. One informant replied:

A mother should look after her child when it's sick. Take it to the clinic. Otherwise, everyone in a house should care for the child, wash it and clean it up. Other people look after children if the mother is tired.

This view of shared responsibility for young children was repeated on a number of occasions. Non-Aboriginal key informants reported they commonly saw young children, who were mobile and no longer exclusively breast-fed, being cared for by older siblings or extended family members. The elderly female Aboriginal key informant stated:

Everyday the big kids look after the little kids. I help them look after children. We live together, share looking after the kids.

All Aboriginal key informants stated that all members of the household were responsible for teaching little kids how to use the toilet properly. The maternal and child health nurse (non-Aboriginal) who had lived in the community for four years and another non-Aboriginal key informant who had lived in the community for over 30 years, both said that they had never observed or heard about efforts by carers to toilet train children.

Aboriginal key-informants believed hosing or using the tap was the best way to clean faeces off a young child. Two non-Aboriginal key informants reported they frequently saw primary school aged children providing this service for younger children. The Aboriginal pre-school teacher reported that most children, when starting at pre-school at approximately four years of age, know very little about hygiene. The pre-school teachers, he believes, introduce young children to good hygiene practice for the first time. This includes how to use the toilet properly and wash their hands with soap, not to spit, and not to wipe the discharge from their noses on their clothing. He believes that there are no positive role models for these children at home, and parents do not reinforce what the children learn at school.

## Discussion

A number of physical barriers were identified that discouraged householders from practising safe hygiene. Significant among these barriers was the non-function of health hardware items (for example - shower, shower stalls, taps, toilets, sink/basins, drains). In approximately 64% of cases, an effective repairs and maintenance program could have prevented this failure of health hardware. In the Uwankara Palyanyku Kanyintjaku (UPK) Review [35] it is reported that approximately 70% of the household items in the houses surveyed needed repair. However, the authors of this report consider that poor initial specification and construction of the items was largely responsible for the high level of non-functioning items. In addition to the poor functional state of health hardware, many houses were visibly contaminated (human or animal faeces, decaying organic matter), and the characteristics of the contamination indicated a moderate to high risk of infection [27]. The factors underlying this risk of infection clearly include both the failure of health hardware and poor hygiene practices.

Our findings indicate that the environmental intervention most urgently required is additional housing with robust essential health hardware. However, while additional housing will reduce some of the social and health problems caused by high levels of household crowding, it will not necessarily resolve the problem of unsanitary living conditions and high rates of infection. The extent that reduced household crowding or improvements in infrastructure will achieve improved health is limited if inappropriate domestic and personal hygiene practices continue unchanged [36,37]. This points to the widely recognised need for, and current efforts to implement "whole- of-government" or inter-sectoral approaches to improve the living conditions in these communities. However, there is no good evidence at this stage that these efforts have had a positive impact [38] and while it may take some time for any impact to become apparent there is a need for more immediate and focussed efforts to improve circumstances for children.

The finding of low levels of knowledge about, and attitudes towards, hygiene behaviour and the transmission mechanisms of common childhood infections is consistent with similar studies in other remote Indigenous communities [39,40]. If participants were aware or alert to the risks, their attitude and behaviour indicated that they did not perceive the risks as serious enough to act on.

The carers of the case study children did show some level of awareness. Their motivation to maintain the health of their children leads them to undertake some protective childcare practices. The high level of support and resources provided by informed extended family members and others provided additional motivation, reinforcing the positive benefits of their behaviours. In this way, their social environment enabled them to sustain their efforts, as the shared values, good support and motivation provided by others prevented them from becoming 'tired' or 'giving up' as other mothers were said to have done. Hubley's Beliefs, Attitudes, Subjective Norms and Enabling Factors (BASNEF) Model of behaviour change emphasises the role played by social norms and peer influences in trying to achieve health behaviour changes among disadvantaged groups [41]. The roles played by existing social norms and peer influence need to be taken into account when planning hygiene improvement programs because population approaches to hygiene improvement are as important as efforts to educate individuals [42]. The failure of these motivated carers of case study children to prevent their children acquiring infections is almost certainly due to the high levels of contamination of the public environment [43]. The immature immune systems of young children and their increasing mobility and independence make it difficult for carers to protect their young children from infection. Such are the levels of environmental contamination that the carers of the case study children would need to take extraordinary measures, going against local social norms, to protect their children from infection.

All the participants in this study appeared to value the image of good hygiene and desired to reduce the levels of infection among children. That the image of good hygiene, or the perceived effects of good hygiene, is positively valued is an essential prerequisite for behaviour change [41]. As the image of good hygiene is already valued, there is a need to focus on developing enabling environments for behaviour change.

This investigation exposed several current childcare practices related to children's hygiene that if not addressed are likely to limit the success of any future hygiene improvement programs. The childcare and parenting practices observed in the study community were compared to those observed in the 1970s by Hamilton [44] and Middleton and Frances [45] in other remote Aboriginal communities not long after groups of Aboriginal people were drawn from their hunter-gatherer lifestyle to live in permanent settlements. We found that childcare practices remain much the same with few changes having occurred over the past 30 - 40 years. Similar to what has occurred in developing countries, the introduction of water and sanitation technology into remote community households has not automatically resulted in changes in childcare practices and hygiene behaviour [46]. Making this comparison, and observations made in more recent studies [39,40,47], highlights that the practices observed in the study community have much in common with different Aboriginal people living in other communities. This is despite these communities being separated by vast distances. The practices identified that continue today include shared mothering, encouraging young children to be independent and to explore and come to terms with their environment, the expectation that mothers will not cause their children distress, and approaches to child care that largely focus on protecting children from physical dangers in the environment and not pathogens. We consider four current childcare practices as especially important barriers to reducing rates of infection among children. However, it needs to be recognised that these childcare practices do offer other social and emotional benefits to children, while the resulting problems are largely due to physical changes in the living environment and larger numbers of people living together in permanent settlements rather than in smaller dispersed family groups.

Firstly, the tradition of sharing responsibility for the day-to-day care of young children, and the degree of freedom very young children have in determining their own care, enables them to reject any hygiene training attempted by their carers. Few carers are appropriately skilled to ensure children comply with their wishes. Secondly, children are frequently taking responsibility for the hygiene needs of infants and toddlers but there are few positive role models for them to emulate. In addition, the physical and social home environment is often not conducive to carrying out the most basic of good hygiene practices. Thirdly, the generally accepted practice of young children defaecating in the open, and the general acceptance of children's faeces in the environment, is indicative of a high tolerance of unsanitary living conditions [48]. This supports focus group findings whereby participants were found to value the images of good hygiene behaviours but did not appear to relate these to their own behaviours and living conditions. Measurable health improvements are unlikely to occur in remote communities while only a few people adopt safe hygiene practices, and levels of environmental contamination remain high [49]. Lastly, study participants indicated that it was the role of pre-school teachers to introduce young children to the concepts of good hygiene and safe hygiene behaviour and not family members. This is a likely consequence of past assimilation policies whereby mission and government staff assumed responsibility to train children in the habits of "industry, cleanliness and order" [50]. These factors exist in a social context common to remote Aboriginal communities of high levels of family and community dysfunction with significant other threats to children's health and welfare [51]. In planning future hygiene improvement programs it is neither appropriate nor practical to consider trying to suddenly change these childcare practices as negative unintended consequences are likely to result. However, population level programs need to be planned after consulting with community members to identify ways to limit the negative impact of these practices and voluntary changes to health promoting behaviour are achieved.

The findings of this study contribute to understanding the factors that underpin current hygiene behaviours in remote Aboriginal communities. Significant barriers to achieving hygiene improvement in these communities have been described and opportunities for future programs identified. Further research is required in a number of areas. There has been little or no research into cultural beliefs concerning dirt, faeces and common childhood illness that potentially be used to build on cultural understanding and work towards a healthier environment in remote communities. Similarly, there has been little research in how community members utilise their houses, into understanding the social and cultural issues relevant to effective maintenance of community housing, or into models of sustainable community housing repairs and maintenance programs. Without this, failure of essential items of housing infrastructure will continue to be a barrier to improving hygiene in these communities. In the past, in some remote Aboriginal communities, there have been interventions such conducting basic plumbing repairs and maintenance programs for householders and the establishment of communal laundries, supply of industrial rather than domestic washing machines, and the free issue of refrigerators and washing machines to householders. However, in most cases these did not result in improved hygiene and were not sustainable. Unfortunately, such interventions have not been evaluated and the likely reasons for why they failed to make any sustainable improvements in hygiene are not known but surmised.

There is a specific need to identify sustainable strategies for keeping the living environment free of children's faeces. The achievement of significant improvement in hygiene will require a better understanding of Aboriginal parenting and childcare practices. Many of the issues discussed are common to disadvantaged groups in developed and developing country contexts. However, the challenges and opportunities for addressing these issues in a resource rich country like Australia differ somewhat to the situation of less wealthy countries where similarly poor environmental conditions are prevalent. It is important that Australia does not repeat the policy failures, for example - the housing policies that have contributed to the current situation.

## Conclusion

Hygiene improvement programs that aim to reduce the rate of infection among young Aboriginal children are likely to have a very limited impact without addressing the poor state of housing infrastructure. The use of more appropriate technology, and the presence of proactive repairs and maintenance programs, should greatly improve the functioning of household infrastructure. However, other likely causes for non-functioning health hardware, including that of inappropriate householder behaviour, also need attention. There is also a well-recognised need to build additional houses as a means to reduce crowding and transmission of infection. Changing current hygiene behaviour, and creating healthy living environments for children, is more likely to be successful in a physical environment that enables good hygiene behaviour. Key parenting and childcare practices that contribute to the high burden of infection among young children need to be addressed. While this research has explored some important issues related to parenting, the dearth of research in this and other areas provides a limited evidence base for the development of health promotion programs. Significant benefit should flow from good monitoring and evaluation of interventions in this area.

We identified a number of predisposing factors (the failure of health hardware and poor hygiene practices) that are barriers to hygiene improvement and reducing the high burden of infection among the children in the study community. These barriers are common to many remote Aboriginal communities in Central and Northern Australia. Overcoming these barriers presents a challenge to achieving improved hygiene in these communities. However, the general desire of study participants to improve the health of their children, and their own desire for a clean and healthy image, makes the challenge less daunting.

## List of Abbreviations

IHANT: Indigenous Housing Association of the Northern Territory; HICH: Housing Infrastructure and Child Health; MSHR: Menzies School of Health Research; NT: Northern Territory; BASNEF: Beliefs, Attitudes, Subjective Norms and Enabling Factors.

## Competing interests

The authors declare that they have no competing interests.

## Authors' contributions

EM developed the study design, collected and analysed the data and drafted the manuscript. RB provided expert advice and support throughout the study. He had a major role in revising the manuscript. JG provided expert advice concerning qualitative methodologies and participated in editing the manuscript. DB provided expert advice on all issues concerning child health and participated in editing the manuscript. All authors read and approved the final manuscript.

## Pre-publication history

The pre-publication history for this paper can be accessed here:



## Supplementary Material

Additional file 1 **Appendices**. Provides supporting information, for example - copies of data collection forms, protocols, interview check lists, examples of data analysis and growth charts. Click here for file

Additional file 2 **Table S1**. A Table that provides focus group findings, including examples of drawings and focus group responses. Click here for file

## References

[B1] Alderete E (1999). The Health of Indigenous Peoples.

[B2] Miller M, Aramburu C, Holzscheiter A (2003). Ensuring the Rights of Indigenous Children.

[B3] Hertzman C, Power C, Heymann J, Hertzman C, Barer M, Evans R (2006). A Lifecourse Approach to Health and Human Development. Healthier Societies: From analysis to action.

[B4] Li SQ, Guthridge SL, Tursan d'Espaignet E, Paterson BA (2007). From Infancy to Young Adulthood: Health status in the Northern Territory, 2006.

[B5] Patton GC, Goldfeld SR, Pieris-Caldwell I, Bryant M, Vimpani GV (2005). A picture of Australia's children. Do we have a clear enough picture to guide rational health and social policy responses?. Med J Aust.

[B6] Brown A, McDonald MI, Calma T (2007). Rheumatic fever and social justice. Med J Aust.

[B7] Maguire GP, Nelson C (2006). Acute rheumatic fever and rheumatic heart disease: an insight into Aboriginal health disadvantage and remote Australia. Med J Aust.

[B8] Ewald DP, Hall GV, Franks CC (2003). An evaluation of a SAFE-style trachoma control program in Central Australia. Med J Aust.

[B9] Taylor HR (2001). Trachoma in Australia. Med J Aust.

[B10] Currie BJ (2002). Rheumatic fever in Aboriginal children (Editorial). J Paediatr Child Health.

[B11] Mak DB (2006). Better late than never: a national approach to trachoma control. Med J Aust.

[B12] Marmot M (2005). Social determinants of health inequalities. Lancet.

[B13] Bailie RS, Runcie MJ (2001). Household infrastructure in aboriginal communities and the implications for health improvement. Med J Aust.

[B14] 2001 Census QuickStats: Kunbarllanjnja-Oenpelli (Indigenous Location). http://www.censusdata.abs.gov.au/ABSNavigation/prenav/ViewData?method=Location%20on%20Census%20Night&subaction=3&producttype=QuickStats&areacode=ILOC3103501&action=401&collection=Census&textversion=true&breadcrumb=PL&period=2001&navmapdisplayed=false&.

[B15] Territory Housing (2000). An analysis of NT regions: CHINS 99.

[B16] Djayhgurrnga EB, Singh JN (1989). The Languages of the People at Oenpelli. Ngoonjook: Batchelor Journal of Aboriginal Education.

[B17] Condon J, Warman G, Arnold L (2001). The Health and Welfare of Territorians.

[B18] Australian Bureau of Statistics, Australian Institute for Health and Welfare (2008). The Health and Welfare of Australia's Indigenous People 2008.

[B19] d'Espaignet E, Paterson B, Kennedy K, Measey M (1998). From Infancy to Young Adulthood - Health Status in the Northern Territory.

[B20] Australian Bureau of Statistics (2007). Health of Children in Australia: A Snapshot, 2004-05.

[B21] Northern Territory Department of Health and Community Services (2003). Growth Assessment and Action Program: GAA Data Collection April 2003.

[B22] World Health Organization (1979). Measurement of Nutritional Impact.

[B23] Population Characteristics, Aboriginal and Torres Strait Islander Australians, 2001. http://www.abs.gov.au/Ausstats/abs@.nsf/0/4B01828AAE8653CDCA256DCE007F78E2?Open.

[B24] National Aboriginal and Torres Strait Islander Health Council (1989). The National Aboriginal Health Strategy.

[B25] Australian Institute of Health and Welfare (AIHW) (2002). Australia's Health No 8.

[B26] Bailie RS, Stevens MR, McDonald E, Halpin S, Brewster D, Robinson G, Guthridge S (2005). Skin infection, housing and social circumstances in children living in remote Indigenous communities: testing conceptual and methodological approaches. BMC Public Health.

[B27] International Scientific Forum on Home Hygiene (2002). Guidelines For Prevention of Infection and Cross Infection in the Domestic Environment - Focus on home hygiene issues in developing countries.

[B28] Fishbein M, Ajzen I (1975). Belief, Attitude, Intention, and Behaviors.

[B29] Green LW, Kreuter MW (1999). Health promotion planning: an educational and ecological approach.

[B30] Curtis V, Cairncross S, Yonli R (2000). Domestic hygiene and diarrhoea - pinpointing the problem. Trop Med Int Health.

[B31] Srinivasan L (1993). Tools for Community Participation: A manual for training trainers in participatory techniques.

[B32] Almedom A, Blumenthal U, Manderson L (1997). Hygiene Evaluation Procedures: Approaches and Methods for Assessing Water and Sanitation Related Hygiene Practices.

[B33] Berggren WL, Wray JD (2002). Positive deviant behavior and nutrition education. Food Nutr Bull.

[B34] Australian Bureau of Statistics (2000). 1999 Australian Housing Survey: Housing Characteristics, Costs and Conditions.

[B35] Nganampa Health Council, SA Health Commission, Aboriginal Health Organisation SA (1987). Report of Uwankara Palyanyku Kanyintaku: An Environmental and Public Health Review within the Anangu Pitjantjatjara Lands.

[B36] Aiello AE, Larson EL (2002). What is the evidence for a causal link between hygiene and infections?. Lancet Infect Dis.

[B37] Esrey SA, Potash JB, Roberts L, Shiff C (1991). Effects of improved water supply and sanitation on ascariasis, diarrhoea, dracunculiasis, hookworm infection, schistosomiasis, and trachoma. Bull World Health Organ.

[B38] Morgan Disney and Associates (2006). Synopsis Review of the COAG Trial Evaluations: Report to the Office of Indigenous Policy Coordination.

[B39] Ratnaike RN, Collings MT, Ratnaike SK, Brogan RM, Gibbs A (1988). Diarrhoeal disease: knowledge, attitudes and practices in an aboriginal community. Eur J Epidemiol.

[B40] Skov S (1994). Childhood Diarrhoea in a Central Australian Aboriginal community: Aboriginal Beliefs and Practices in the Context of the Ecology of Health. PhD Thesis.

[B41] Hubley J (1993). Communicating Health: An action guide to health education and health promotion.

[B42] Goldman N, Pebley AR, Beckett M (2001). Diffusion of ideas about personal hygiene and contamination in poor countries: evidence from Guatemala. Soc Sci Med.

[B43] Roundy RW, Roundy LM, Nawalinski T, McGlashan ND, Blunden JR (1983). Scale in the relationship between behaviour and disease. Geographical aspects of health - Essays in honour of Andrew Learmonth.

[B44] Hamilton A (1981). Nature and Nurture, Aboriginal Child-rearing in North-central Arnhem Land.

[B45] Middleton MR, Francis SH (1976). Yuendumu and its Children: Life and health on an Aboriginal settlement.

[B46] Cairncross S (2003). Editorial: Water supply and sanitation: some misconceptions. Trop Med Int Health.

[B47] Tynan BJ (1979). Medical Systems in Conflict: A Study of Power. PhD Thesis.

[B48] Curtis V, Kanki B, Mertens T, Traore E, Diallo I, Tall F, Cousens S (1995). Potties, pits and pipes: explaining hygiene behaviour in Burkina Faso. Soc Sci Med.

[B49] Cairncross S, Blumenthal U, Kolsky P, Moraes L, Tayeh A (1996). The public and domestic domains in the transmission of disease. Trop Med Int Health.

[B50] Attwood B, Read P (2000). Space and Time at Ramahyuck, Victoria, 1863-85. Settlement: A History of Australian Indigenous Housing.

[B51] Sutton P (2001). The politics of suffering: Indigenous policy in Australia since the 1970s. Anthropological Forum.

