# Supplementary material for: A case study of physical and social barriers to hygiene and child growth in remote Australian Aboriginal communities
Source: BMC Public Health. 2009 Sep 18;9:346. doi: 10.1186/1471-2458-9-346 (PMC2758870; doi:10.1186/1471-2458-9-346)
Supplement: Additional file 1 — Appendices. Provides supporting information, for example - copies of data collection forms, protocols, interview check lists, examples of data analysis and growth charts. [file 1471-2458-9-346-S1.DOC]

# Additional File

Appendices

1. Housing Survey Form

2. Housing Infrastructure Data Collection Form

3. Sample Picture Card

4. 3-Pile Sorting Focus Group Protocol

5. Example - Formatted Notes from One Focus Group

6. Example - Summary of Focus Group Findings

Example Card: Card A6. Cards were printed on A4 size paper and laminated for ease in handing around**.** Several were coloured but most were in black and white.

7. Copies Growth Charts for Case Study Children

8. Case Study Interview Check List

Housing Survey Form


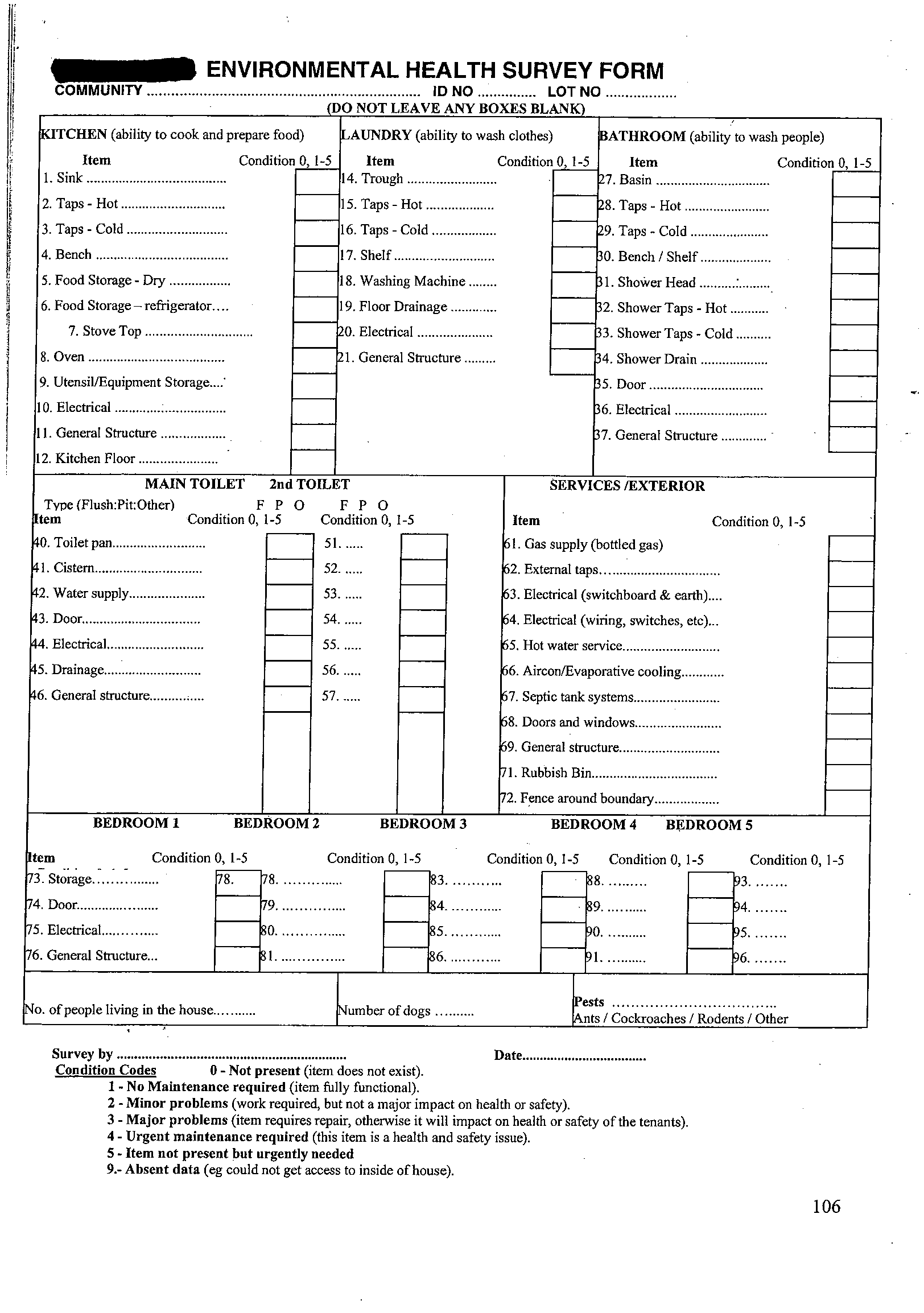


Housing Infrastructure Data Collection Form

**Data Collection Form Housing Infrastructure Focus Group Exercise**

| **Category**  **Nos.** | **Definition** |
| --- | --- |
|  | Malicious damage is intentional damage as a result of anger, attention seeking, retribution, conflict, intoxication, substance abuse, etc. |
|  | Damage is caused by normal wear and tear and lack of maintenance over time, i.e. the damage is not the fault of the tenants. |
|  | Inappropriate use is the use of items for an intention other than what the item was designed for, e.g. toilet blocked by disposable nappies or other inappropriate matter. |
| 5 | Neglect is the failure to care for items or materials such that serious damage occurs, e.g. water leakages over a prolonged period of time causing dampness and rot. |
| 6 | The technology in place cannot be expected to withstand the conditions or degree of usage of the household. |
| 7 | Poor design, use of inferior materials or inferior workmanship is responsible for damage. |
| 8 | More than 1 category applies. |
| 9 | It is not possible to identify the probable cause of damage. |

**Lot No:………… Year built……… Capital Work ………**

Nos Bedrooms……… Nos Adults………. Nos Child……....

| **Item** | **Description** | **Catergory Number** | **Cause**  **1** | **Cause**  **2** | **Comment** |
| --- | --- | --- | --- | --- | --- |
|  | **Kitchen** |  |  |  |  |
| 1 | Sink |  |  |  |  |
| 2 | Taps - Hot |  |  |  |  |
| 3 | Taps - Cold |  |  |  |  |
| 4 | Bench |  |  |  |  |
| 5 | Food Storage - dry |  |  |  |  |
| 6 | Food Storage - Ref |  |  |  |  |
| 7 | Stove Top |  |  |  |  |
| 8 | Oven |  |  |  |  |
| 9 | Utensil Storage |  |  |  |  |
| 10 | Electrical |  |  |  |  |
| 11 | General Structure |  |  |  |  |
| 12 | Kitchen Floor |  |  |  |  |
|  | **Laundry** |  |  |  |  |
| 14 | Trough |  |  |  |  |
| 15 | Taps-Hot |  |  |  |  |
| 16 | Taps-Cold |  |  |  |  |
| 17 | Shelf |  |  |  |  |
| 18 | Washing Machine |  |  |  |  |
| 19 | Floor Drainage |  |  |  |  |
| 20 | Electrical |  |  |  |  |
| 21 | General Structure |  |  |  |  |
|  | **Bathroom -1** |  |  |  |  |
| 27.1 | Basin |  |  |  |  |
| 28.1 | Taps-Hot |  |  |  |  |
| 29.1 | Taps-Cold |  |  |  |  |
| 30.1 | Bench/Shelf |  |  |  |  |
| 31.1 | Shower Head |  |  |  |  |
| 32.1 | Shower Taps-Hot |  |  |  |  |
| 33.1 | Shower Taps-Cold |  |  |  |  |
| 34.1 | Shower Drain |  |  |  |  |
| 35.1 | Door |  |  |  |  |
| 36.1 | Electrical |  |  |  |  |
| 37.1 | General Structure |  |  |  |  |
|  | **Bathroom - 2** |  |  |  |  |
| 27.2 | Basin |  |  |  |  |
| 28.22 | Taps-Hot |  |  |  |  |
| 29.2 | Taps-Cold |  |  |  |  |
| 30.2 | Bench/Shelf |  |  |  |  |
| 31.2 | Shower Head |  |  |  |  |
| 32.2 | Shower Taps-Hot |  |  |  |  |
| 33.2 | Shower Taps-Cold |  |  |  |  |
| 34.2 | Shower Drain |  |  |  |  |
| 35.2 | Door |  |  |  |  |
| 36.2 | Electrical |  |  |  |  |
| 37.2 | General Structure |  |  |  |  |
|  | **Toilet -1** |  |  |  |  |
| 40 | Toilet Pan |  |  |  |  |
| 41 | Cistern |  |  |  |  |
| 42 | Water Supply |  |  |  |  |
| 43 | Door |  |  |  |  |
| 44 | Electrical |  |  |  |  |
| 45 | Drainage |  |  |  |  |
| 46 | General Structure |  |  |  |  |
|  | **Toilet-2** |  |  |  |  |
| 51 | Toilet Pan |  |  |  |  |
| 52 | Cistern |  |  |  |  |
| 53 | Water Supply |  |  |  |  |
| 54 | Door |  |  |  |  |
| 55 | Electrical |  |  |  |  |
| 56 | Drainage |  |  |  |  |
| 57 | General Structure |  |  |  |  |
|  | **Services/Exterior** |  |  |  |  |
| 63 | Electrical (S/board&earth) |  |  |  |  |
| 64 | Electrical (Wiring/Switches) |  |  |  |  |
| 65 | Hot water service |  |  |  |  |
| 71 | Rubbish Bin |  |  |  |  |
| 72 | Fence |  |  |  |  |
|  |  |  |  |  |  |
|  | **Other** | **Contaminants** | | |  |
| 73 | Kitchen Bench Top |  | | |  |
| 74 | Internal Floor |  | | |  |
| 75 | External Floor |  | | |  |

Signed: 1..……….. 2…………

3………… 4………….

Date: ………………

Picture Card

All cards were A4 size and laminated for easy handling by a large number of people.


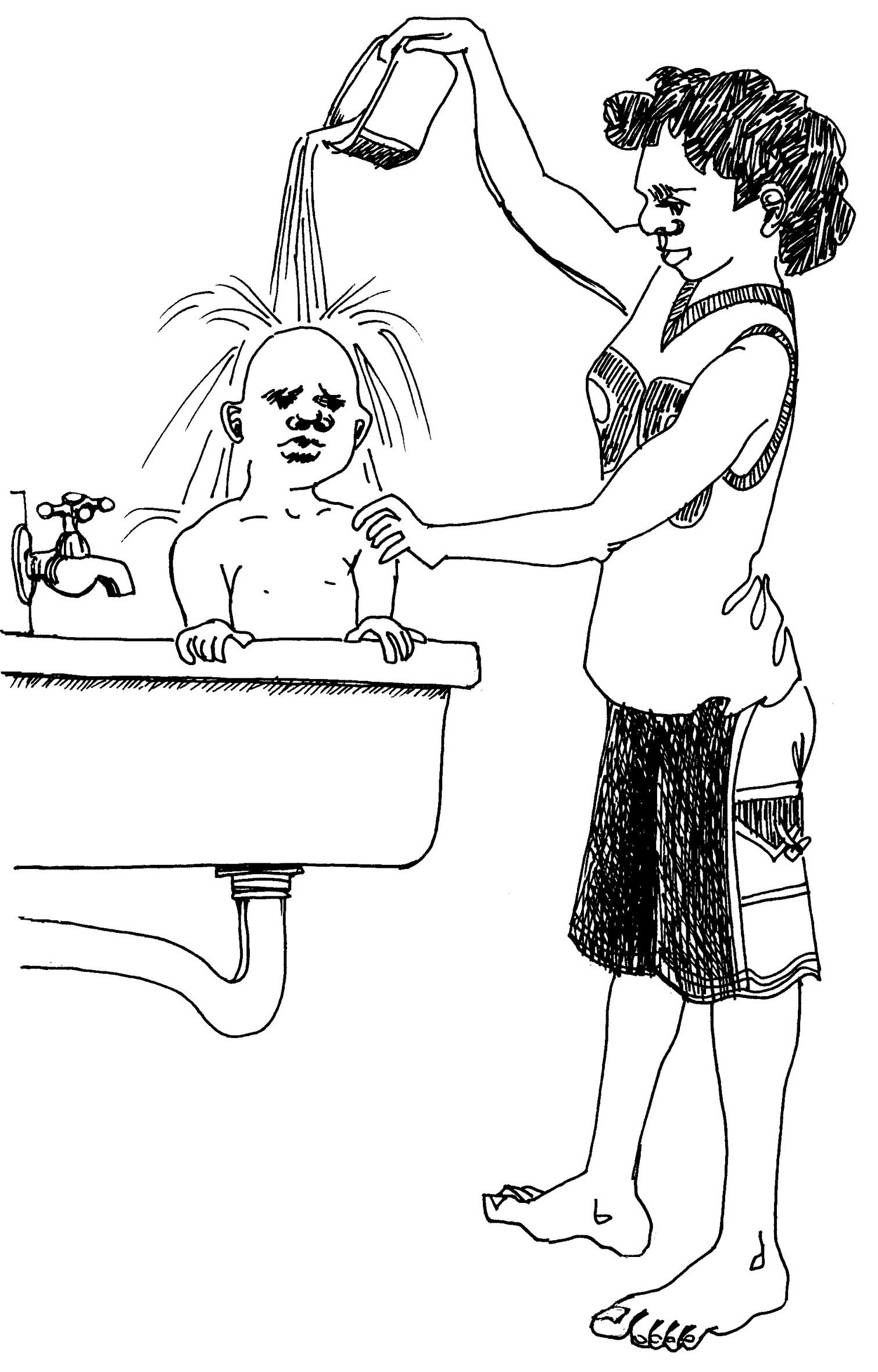


Focus Group Protocol

INTRODUCTION TO 3 PILE SORTING EXERCISE

*Thank you for agreeing to talk to me about kids’ health. What I am going to do is pass around one card at a time for everyone to look at and talk about. When you are ready I will need someone to tell me what is happening in the picture. If you are not sure what is happening in the picture please ask me questions about the picture. We do this to make sure we are all talking about the same things. When you have finished talking to each other about what is happening in the picture someone should put the picture next to one of these 3 signs, ‘good’, ‘not good’, ‘not sure’.*

*Put the picture near the ‘good’ sign if you think that the story the picture tells you is healthy, put it near the ‘not sure’ sign if you cannot decide or if you are not sure. Put the card near the ‘not good’ sign if you think what is happening in the picture is not healthy. Don’t worry about making mistakes, there are no perfect right or wrong answers. I am just interested in knowing what you think about these things.*

*After the group has looked at all the cards, I will ask someone to tell me the story about why you made this decision so I can understand better about what the people in this community think and feel about these things.*

Record of Focus Group Discussion

Formatted Notes from One Focus Group

| **A1** |
| --- |
| Feedback  **Good** |
| Reason  Two kids healthy. Eating bush tucker. Tell by looking at them. |
| **A2** |
| Feedback  **Not Good** |
| Reason  Sores, skinny. Family didn’t look after them. Not good – no decent clothes. Not eating good food. Q. How did he get those sores? A. Sores from eating wrong food. |
| **A3** |
| Feedback  **Not Sure** (Thompson directed how picture should be passed around, i.e. who should see it first) |
| Reason  Dog eating food and there are flies around. Dog eating rubbish/food and make the baby sick. Kid might touch the dog and the dog spread the germs. Wrong place for dog to eat right in front of families. Should be away from families. (It is not clear if the men did not recognize the ‘kimbie’ or using the words rubbish or food because too embarrassed to say it was a kimbie or nappy.) |
| **A4** |
| Feedback  People sitting there and cooking something. **Good** |
| Reason  Family probably eating bush tucker. Looking after family. That’s the way to go. Eating bush food. Teaching young ones how to cook. |
| **A5** |
| Feedback  **Not sure** |
| Reason  Might have diarrhoea in the wrong place. Q. Where is the right place? A. The right place is in the toilet. |
| **A6** |
| Feedback  **Good** |
| Reason  Family sitting under a tree. |
| **A7** |
| Feedback  **Good** |
| Reason  Washing hands. Been to toilet and washing hands. Get the rubbish out. |

Comment: Initially there was a large group of men willing to participate. These were the male artists at the Art Centre. They were impatient to start. Leader directed our activities. On passing the first picture around one man started to speak. What he said (I’ve been to school in Darwin) seemed out of context for what was happening. Many of the men seem to be cross with him and left the group (? Embarrassed). This man continued to repeat over and over the same unrelated things. One of the men sent him away. This left the group with 4 men. Some of the men drifted back and would participate for a short while and leave again. This happened continuously with 4 men being constant. The men were quick and decisive in making their choices. The men agreed in their responses but they did not necessarily seek consensus. Two men spoke out confidently building on each other’s comments. Communicated well in English. Impatient with questioning so did not ask them DOB or level of education. Would appear to have education level of upper primary school. At the end of the session men rushed for gifts. Very much liked body spray but also keen to take soap.

Summary of Focus Group Findings

Example Card: Card A6. Cards were printed on A4 size paper and laminated for ease in handing around**.** Several were coloured but most were in black and white.

Responses from various focus groups for the same card (Good, Not Sure, Not Good)

C5


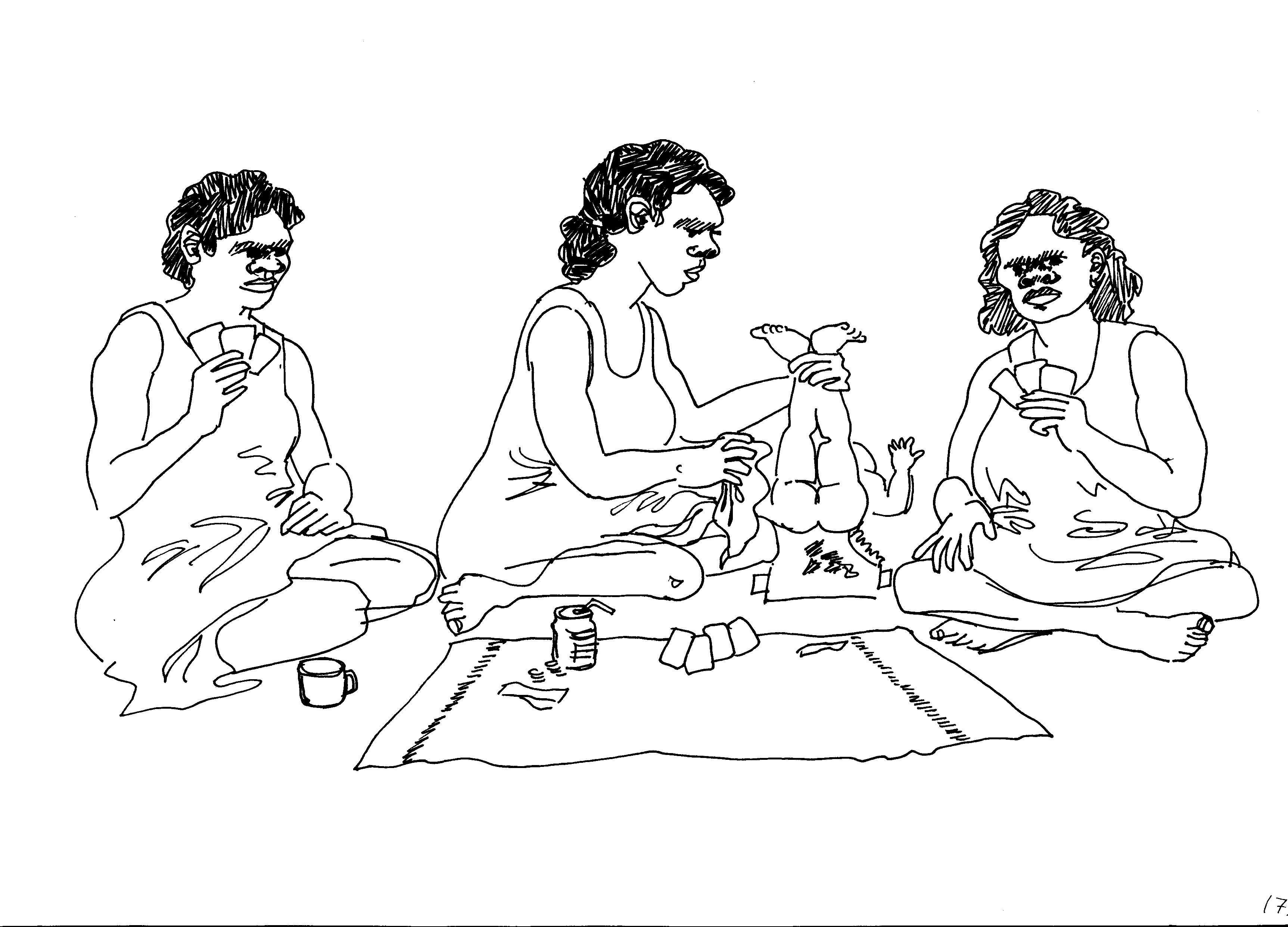


| **Good** | **Not Sure** | **Not Good** |
| --- | --- | --- |
| FG13 Mother-one doing the right thing |  | FG3. They are playing cards with a baby. Baby watching them playing cards. Not giving baby food or milk (Older woman who had been silently participating to this time stated this.) |
|  |  | FG5. Shouldn’t be wasting money, she should be looking after her kids. |
|  |  | FG8. Q. Are they playing cards? A. Yes, 3 ladies playing cards. Card games not good. Probably kids feeling hungry and they spend all the money on cards |
|  |  | FG10. These ladies are playing cards and then she’s cleaning the poo. She should be doing it in the laundry. She cannot/shouldn’t do that because the other ladies are there. That’s probably a green can. It’s not right. |

Growth Charts

**Growth Chart Case Study Child 1**


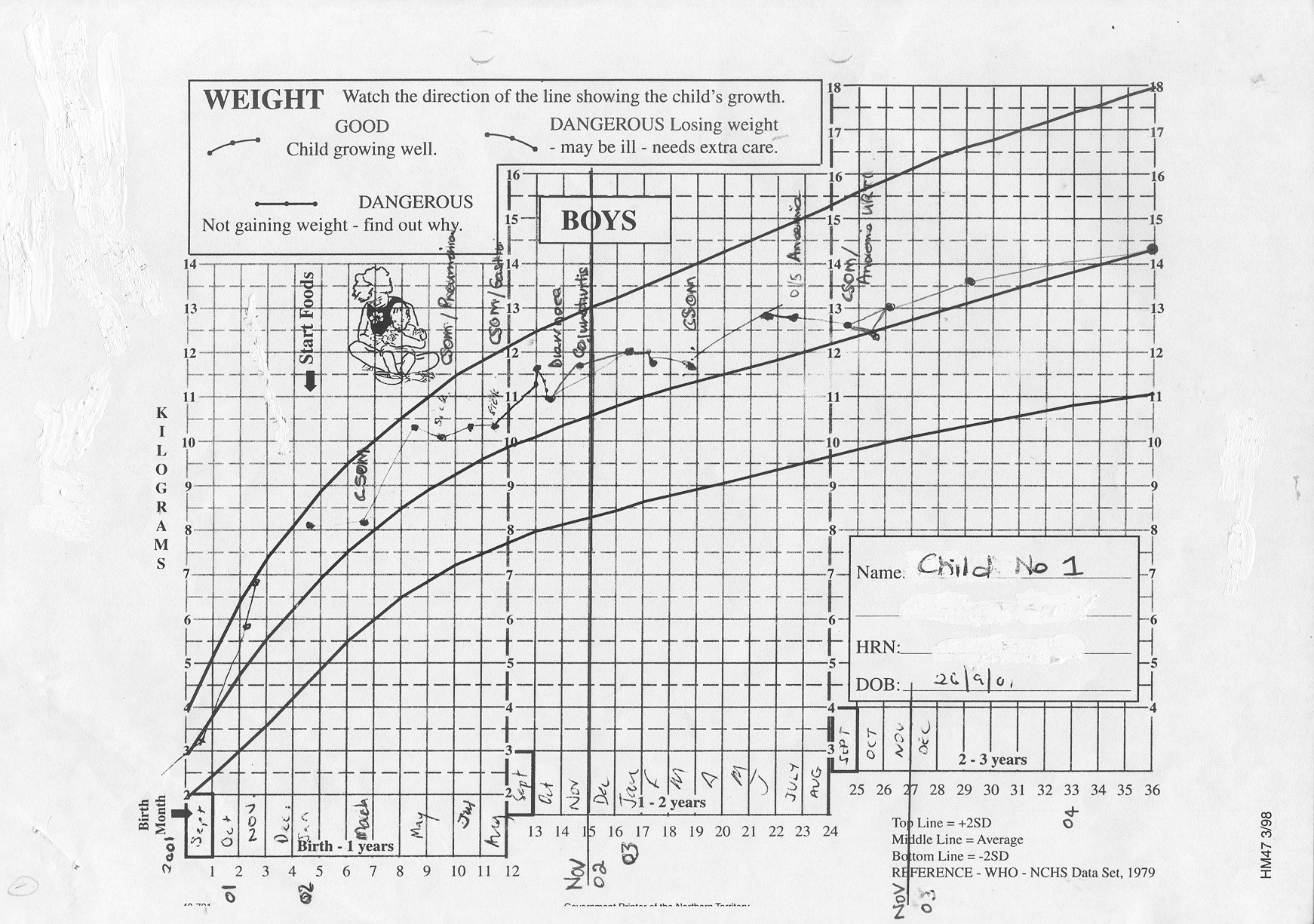


**Growth Chart Case Study Child 2**


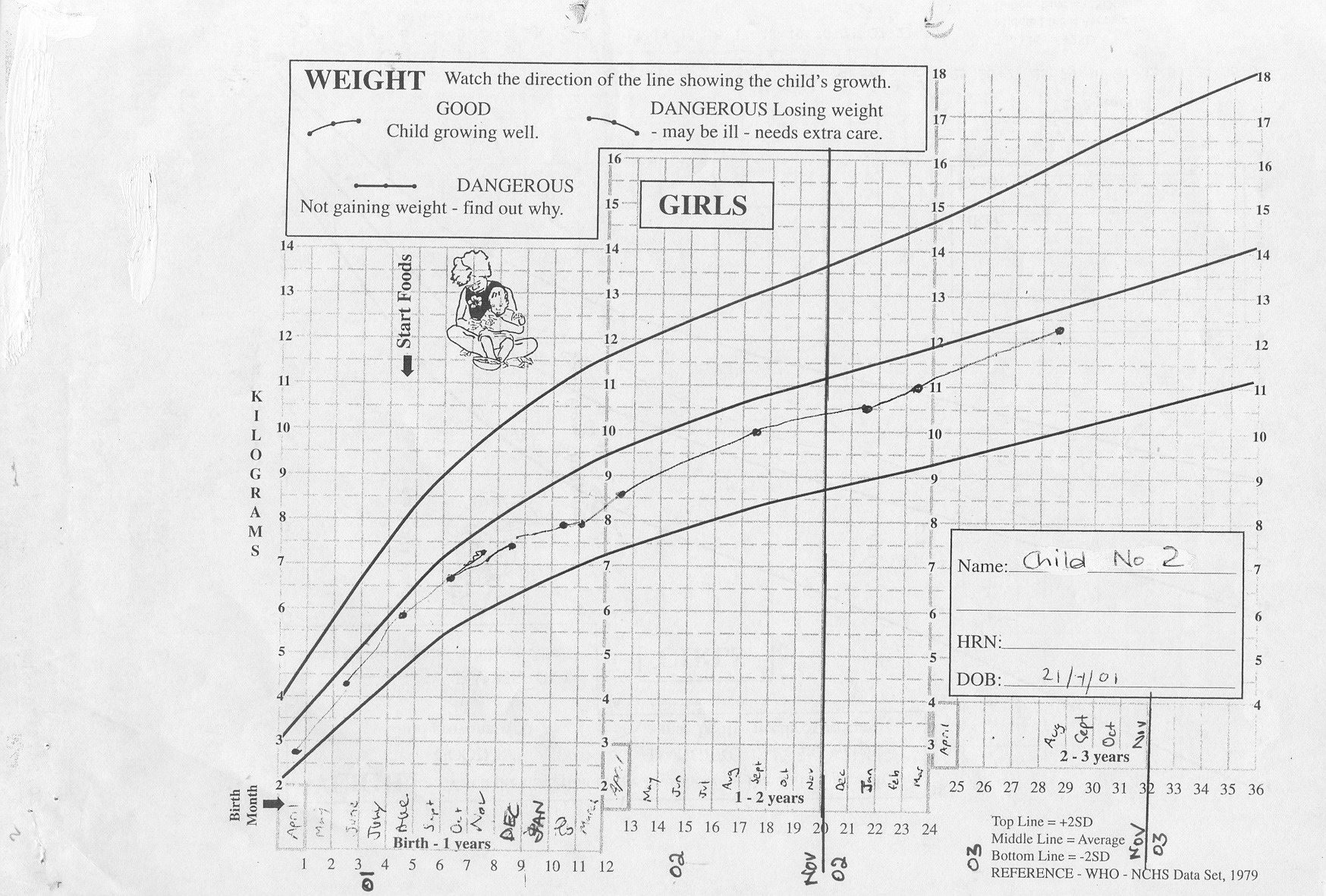


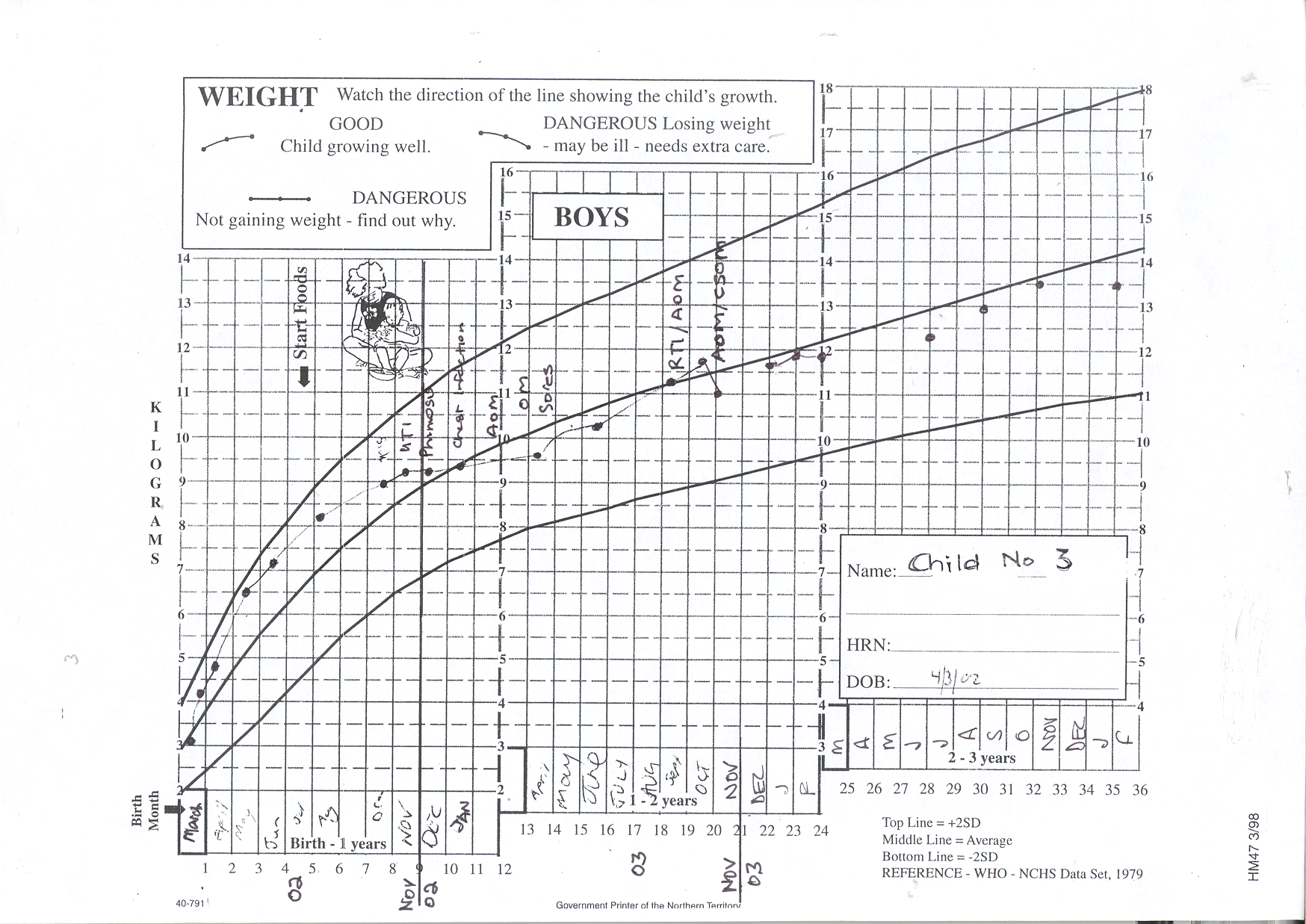


**Growth Chart Cast Study Child 3**

**Growth Chart Case Study Child 4**


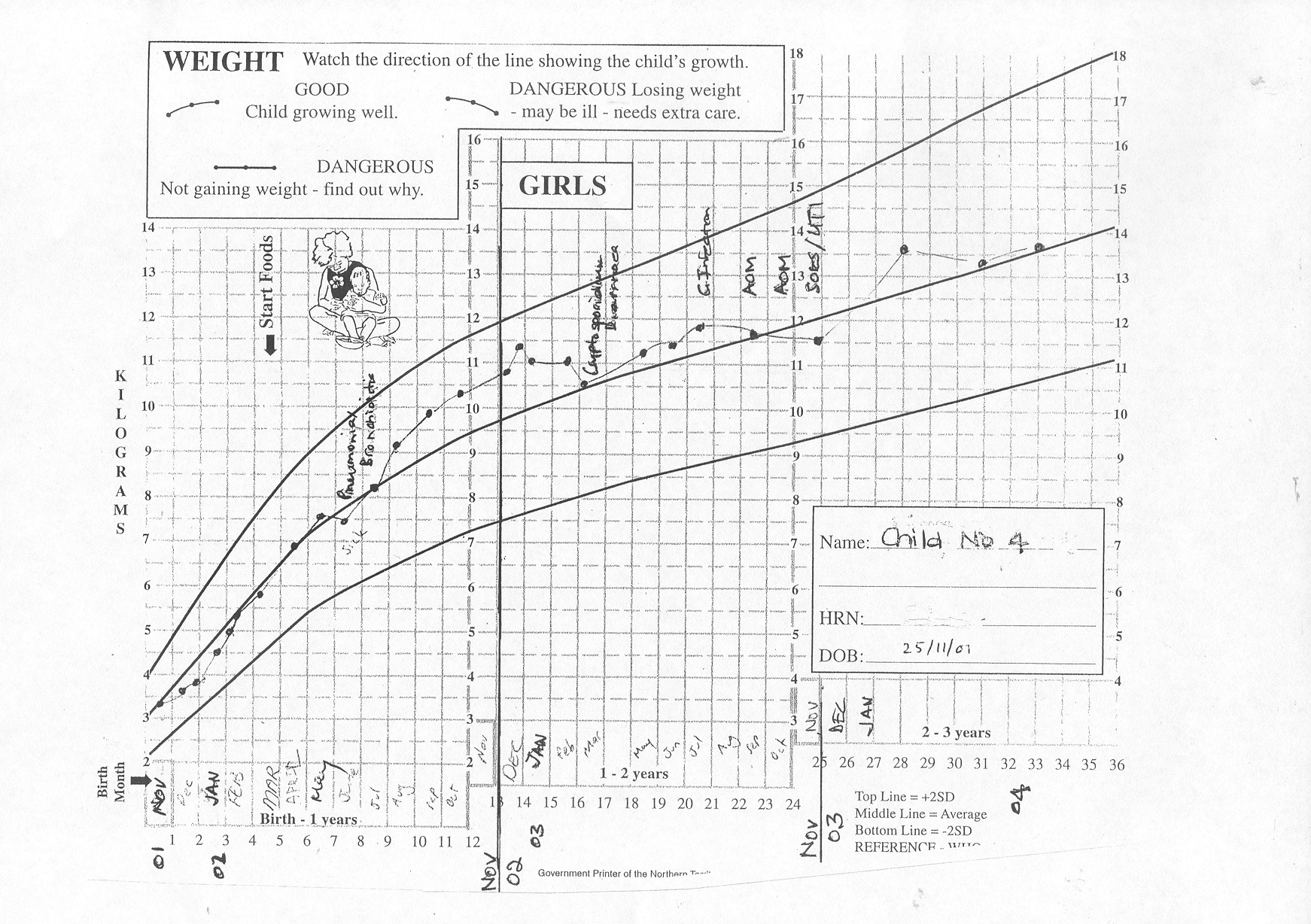


**Growth Chart Case Study Child 5**


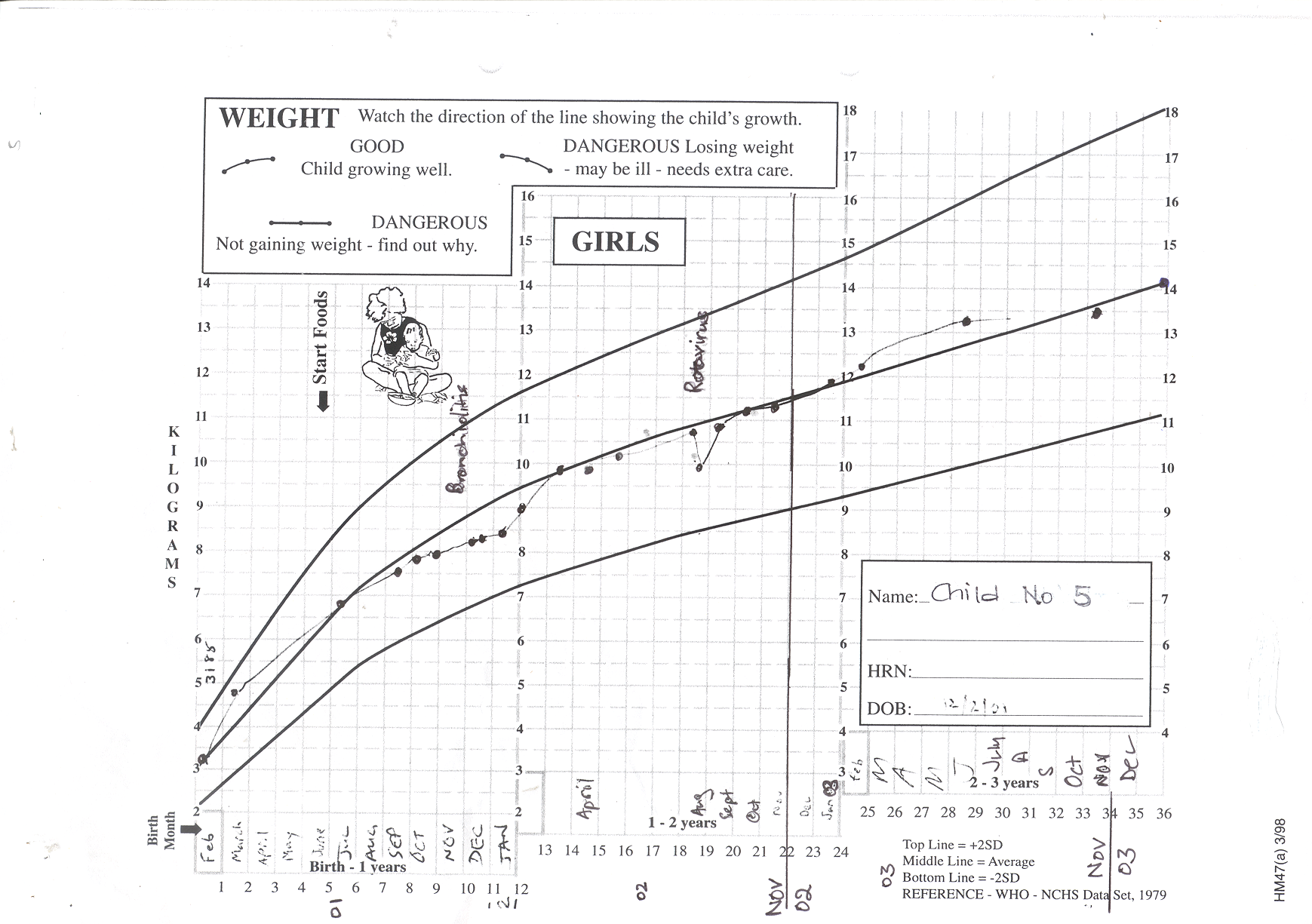


Case Study Interview Check List

Case Study Interview Check List – Points to cover

Child

- Does the child live in same house?
- Who cares for the child?
- Nutrition – type of food, availability, access to food
- Hygiene behaviour (any toilet training/where do they bath the child?)
- Does the child go to the child care centre?
- Place of child in the family – any siblings?
- Sleeping arrangements
- Worry about the child’s health at all?
- Had scabies?

Mother

- Single or has a partner?
- How many children?
- Education level, where did they go to school?
- Income
- Support Systems (Formal and Informal, who provides advice?)
- Health seeking behaviour
- What barriers are present that hinder her caring for her child
- Belong to traditional owner’s family
- Social behaviours (alcohol, smoking, drugs, gambling, active church goer)
- What does she do to stop her children from becoming sick (getting scabies)?

Household

- Who lives in the house?
- How many live in the house?
- What problems are there? (social, infrastructure)
- Relationship of people to each other
- Taps – are handles removed?
- Soap readily available? Affordable?
- Hot water available?
- Is the oven used?
- Is there washing machine or fridge? Do they work ok?
- Who cleans the house?
- Do they have broom, mop or other cleaning aids?
