# Supplementary material for: A case study of physical and social barriers to hygiene and child growth in remote Australian Aboriginal communities
Source: BMC Public Health. 2009 Sep 18;9:346. doi: 10.1186/1471-2458-9-346 (PMC2758870; doi:10.1186/1471-2458-9-346)
Supplement: Additional file 2 — Table S1. A Table that provides focus group findings, including examples of drawings and focus group responses. [file 1471-2458-9-346-S2.DOC]

Table 1. Example of drawing and focus group responses

| Card Group and Number | Card | Theme | Good | Not Sure | Not Good | Number of Focus Groups |
| --- | --- | --- | --- | --- | --- | --- |
| A4 | 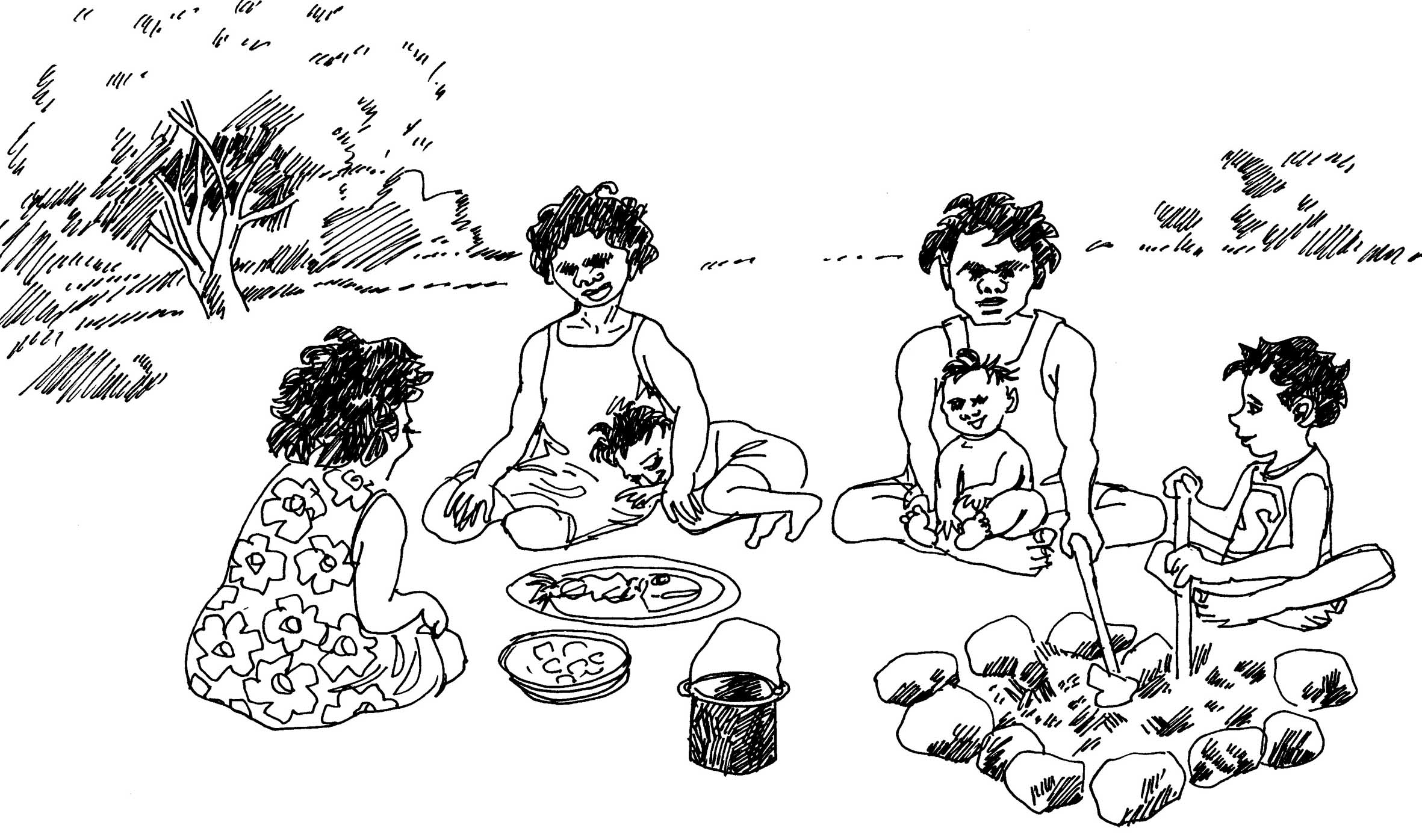 | Healthy living practice | 7 | 0 | 0 | 7 |
| C2 | 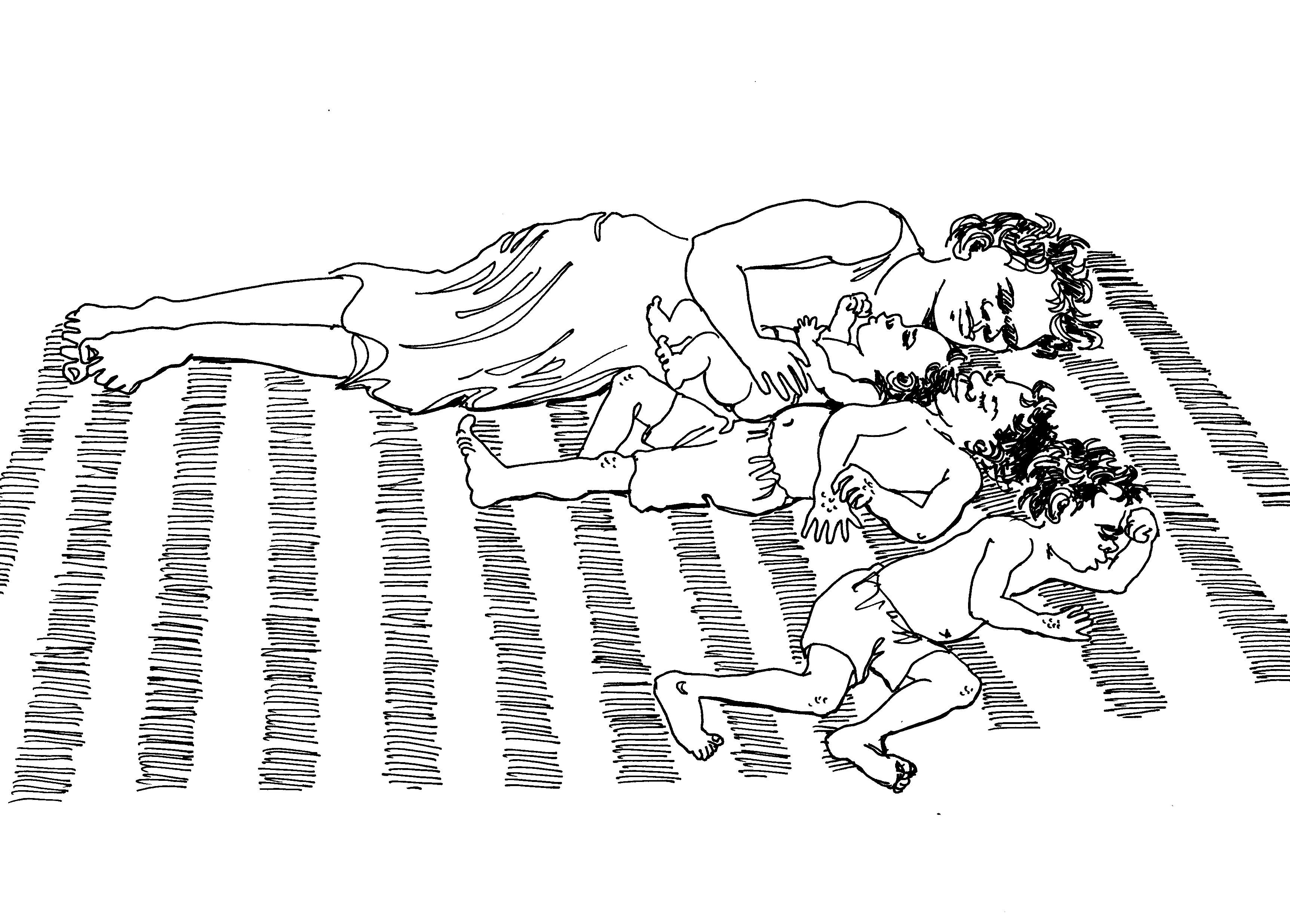 | Skin Infection | 3 | 2 | 0 | 5 |
| B5 | 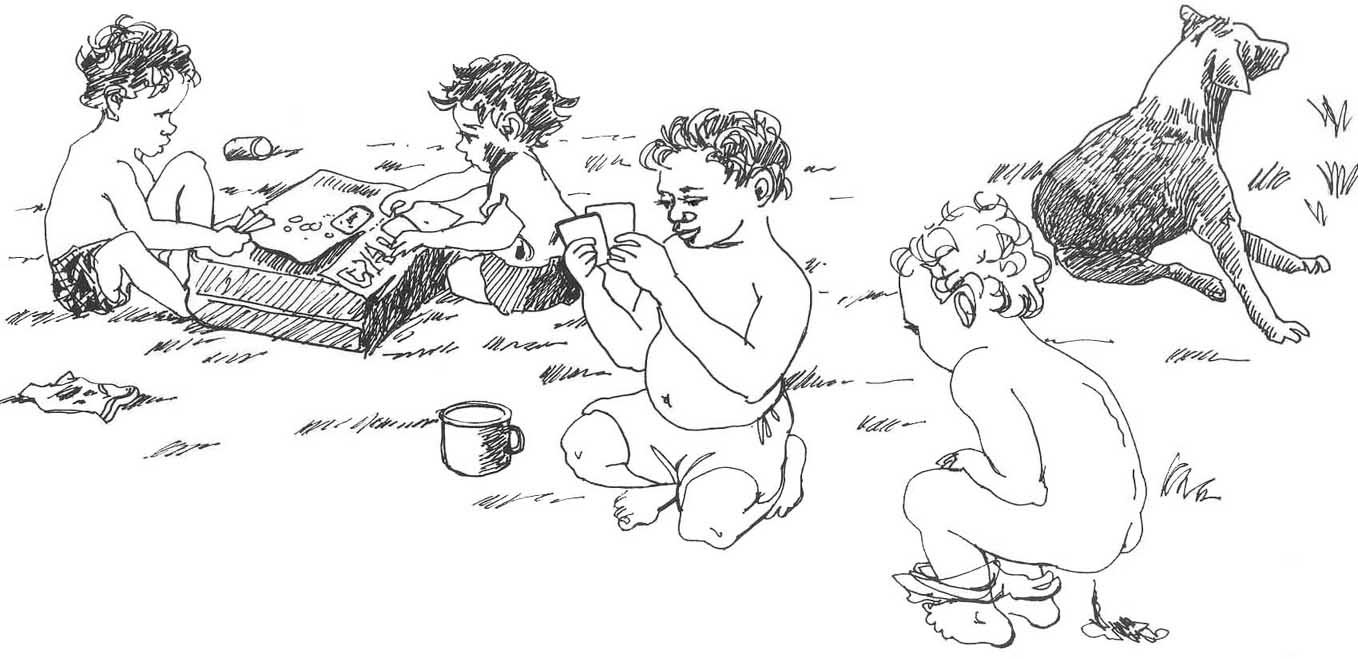 | Diarrhoeal Disease | 0 | 0 | 5 | 5 |
| B6 | 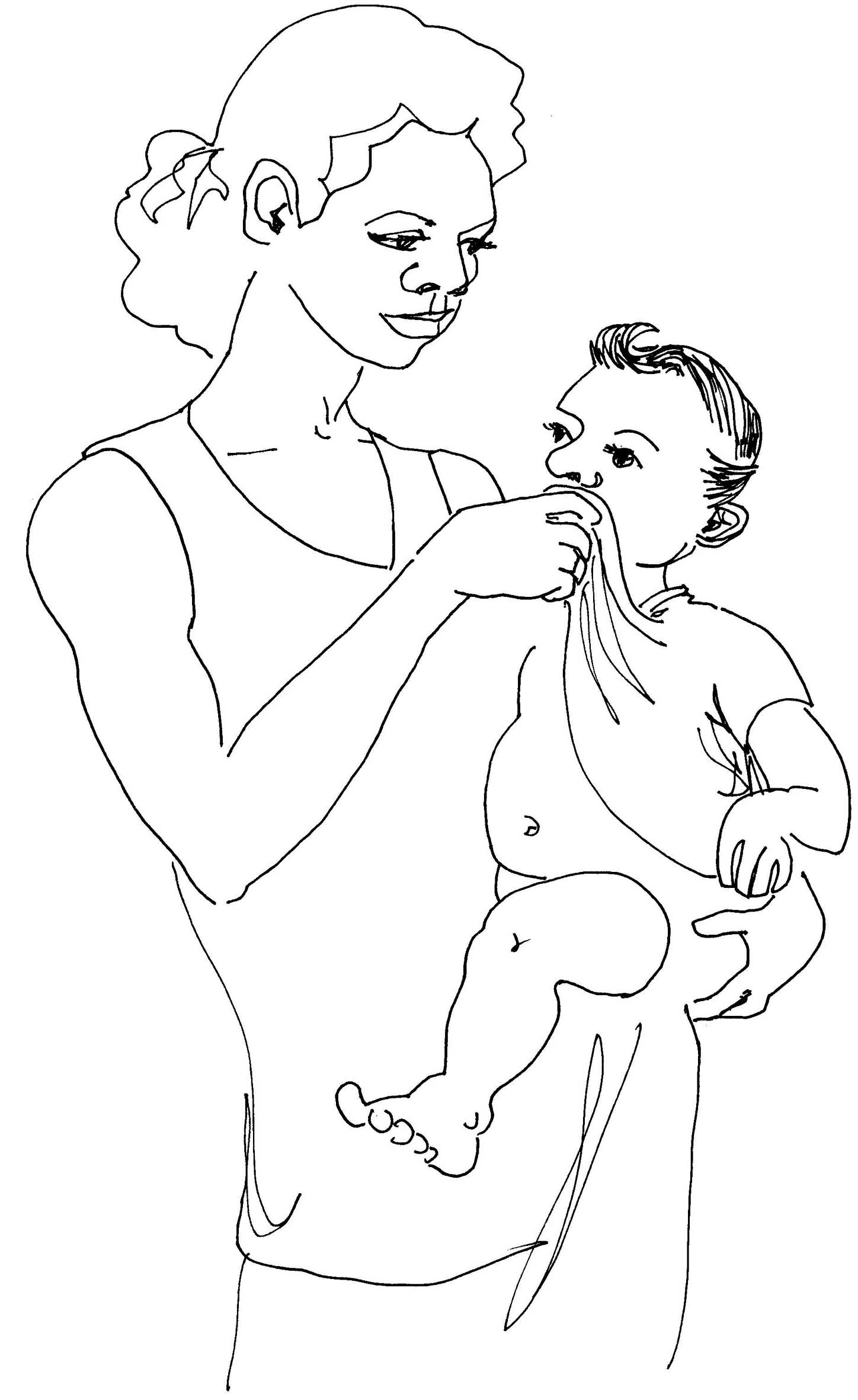 | Respiratory Disease | 1 | 1 | 3 | 5 |
| B7 | 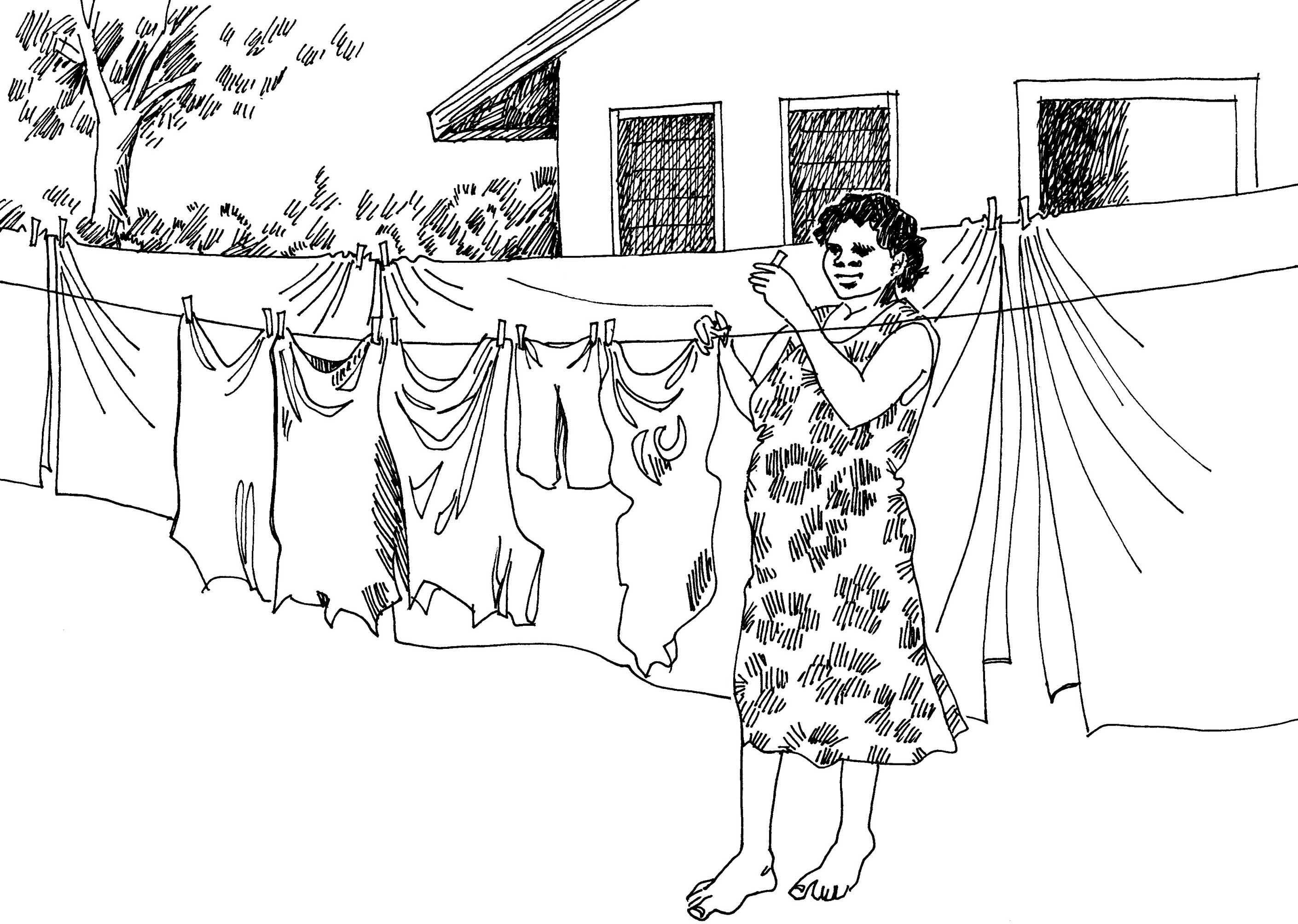 | Healthy living practice | 5 | 0 | 0 | 5 |
